# Phylogenetic significance of the characteristics of simple sequence repeats at the genus level based on the complete chloroplast genome sequences of Cyatheaceae

**DOI:** 10.1002/ece3.8151

**Published:** 2021-09-23

**Authors:** Ming Zhu, Peipei Feng, Jingyao Ping, Jinye Li, YingJuan Su, Ting Wang

**Affiliations:** ^1^ College of Life Sciences South China Agricultural University Guangzhou China; ^2^ School of Life Sciences Sun Yat‐sen University Guangzhou China; ^3^ Research Institute of Sun Yat‐sen University in Shenzhen Shenzhen China

**Keywords:** chloroplast SSR, Cyatheaceae, genus, phylogeny

## Abstract

The simple sequence repeats (SSRs) of plant chloroplasts show considerable genetic variation and have been widely used in species identification and phylogenetic relationship determination. Whether chloroplast genome SSRs can be used to classify Cyatheaceae species has not yet been studied. Therefore, the chloroplast genomes of eight Cyatheaceae species were sequenced, and their SSR characteristics were compared and statistically analyzed. The results showed that the chloroplast genome structure was highly conserved (genome size: 154,046–166,151 bp), and the gene content (117 genes) and gene order were highly consistent. The distribution characteristics of SSRs (number, relative abundance, relative density, GC content) showed taxon specificity. The primary results were the total numbers of SSRs and mononucleotides: *Gymnosphaera* (61–67 and 40–47, respectively), *Alsophila* (121–122 and 95–96), and *Sphaeropteris* (102–103 and 77–80). Statistical and clustering analyses of SSR characteristics showed that their distribution was consistent with the recent classification of Cyatheaceae, which divided the eight Cyatheaceae species into three genera. This study indicates that the distribution characteristics of Cyatheaceae chloroplast SSRs can provide useful phylogenic information at the genus level.

## Graphical abstract

Whether chloroplast genome SSRs can be used to classify Cyatheaceae species has not yet been studied. Therefore, the chloroplast genomes of eight Cyatheaceae species were sequenced by our team, and their SSR characteristics were compared and statistically analyzed. Statistical and clustering analyses of SSR characteristics showed that their distribution was consistent with the recent classification of Cyatheaceae, which divided the eight Cyatheaceae species into three genera.

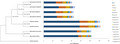

## 1 INTRODUCTION

Simple sequence repeats (SSRs), also known as microsatellites, are short tandem repeat sequences with a motif length of 1–6 bp characterized by high variability and codominant inheritance and have been widely used in species identification, genetic diversity studies, and phylogenetic relationship determination (Chmielewski et al., 2015; Dashnow et al., 2015). SSRs are caused by slipped strand mispairing and subsequent errors during DNA replication, repair, and recombination (Levinson & Gutman, 1987). SSRs are mainly found in intergenic and noncoding regions, with a few present in introns (Li et al., 2004; Liu et al., 2021; Su et al., 2018). Previous studies have shown that the characteristics of genomic SSRs in different taxa (such as their distribution patterns) reflect their phylogenetic relationships (Manee et al., 2020; Srivastava et al., 2019).

The distribution of SSRs in some chloroplast (cp) genomes is nonrandom and dominated by mononucleotides, where A/T bases account for the majority (Ellegren, 2004; George et al., 2015; Ren et al., 2021). Some Polypodiaceae species show similar SSR distribution patterns in their cp genomes (Liu et al., 2021). Recently, Ping et al. (2021) analyzed the distribution pattern of *Cupressus* SSRs and found that the distribution patterns in *Cupressus* and *Hesperocyparis* are highly consistent. In addition, according to the proportions of A/T bases and mononucleotides in *Callitropsis funebris*, this species is closer to *Cupressus*. Studies have shown that the number and types of SSRs in cp genomes are conserved within genera, and the types of SSRs differ extensively among genera in Dryopteridaceae (Fan et al., 2021). Furthermore, cpSSRs continue to provide important new clues to explore the phylogeny among lineages.

The Cyatheaceae are an impressive group of ferns because of their arborescent features and a rich number of species, which account for the vast majority of known tree ferns and are mainly distributed in warm and humid tropical and subtropical regions (Korall et al., 2006; Kramer, 1990; PPG I, 2016; Smith et al., 2006). At present, four representative classification systems apply to Cyatheaceae: (a) the Holttum and Edwards system recognizes only one genus (Holttum, 1963); (b) the Tryon system includes six genera (Tryon, 1970); (c) the Lellinger system divides the family into four genera (Lellinger, 1987); and (d) the Pteridophyte Phylogeny Group (PPG I, 2016) system divides Cyatheaceae into three genera. Ching (1978) classified Chinese Cyatheaceae plants into three genera: *Sphaeropteris*, *Gymnosphaera*, and *Alsophila*. On this basis, Xia (1989) treated *Alsophila* and *Gymnosphaera* as subgenera and combined them into *Alsophila*. Recent molecular phylogenetic studies have shown that Cyatheaceae includes four monophyletic groups, namely, *Alsophila*, *Cyathea*, *Gymnosphaera*, and *Sphaeropteris* (Dong & Zuo, 2018; Janssen & Rakotondrainibe, 2008; Korall et al., 2007; Korall & Pryer, 2014), which provides a framework for us to study the phylogenetic significance of the cpSSRs of Cyatheaceae.

In this study, the cp genomes of eight Cyatheaceae species were sequenced, and the distribution and characteristics of their SSRs were compared. The cp genome sequences of these eight species represent the existing cp genome data for Cyatheaceae, covering most Cyatheaceae genera. Our major objectives were to (a) report the complete cp genomes of *A. denticulate* and *A. metteniana*; (b) compare the distribution patterns of the cpSSRs of the eight Cyatheaceae species; and (c) reveal the phylogenetic significance of the SSRs characteristics. Our findings may serve as a foundation for studying the evolutionary cp genomics and phylogeny of Cyatheaceae.

## 2 MATERIALS AND METHODS

### 2.1 Sampling

The leaves of *Gymnospaera denticulata* Baker and *Gymnospaera metteniana* Hance were collected from Nankunshan in Huizhou and the botanical garden of South China Agricultural University in Guangzhou, respectively. The specimens of *Gymnospaera denticulata* Baker and *Gymnospaera metteniana* Hance are stored in the Herbarium of South China Agricultural University (SCAUB; voucher: M Zhu 201910 and M Zhu 201908). The leaves of *Gymnospaera podophylla* Hook and *Gymnospaera gigantea* Wall. ex Hook were collected from the South China Botanical Garden of the Chinese Academy of Sciences in Guangzhou (Liu et al., 2018; Wang et al., 2019b). The leaves of *Alsophila costularis* Baker, *Sphaeropteris brunoniana* (Hook.) R. M. Tryon, and *Sphaeropteris lepifera* (Hook.) R. M. Tryon were collected from the Fairy Lake Botanical Garden of the Chinese Academy of Sciences in Shenzhen (Liu et al., 2020; Wang et al., 2019a; Zhu et al., 2020). The leaves of *Alsophila spinulosa* (Wall. ex Hook.) R. M. Tryon were collected from the Wuhan Botanical Garden of the Chinese Academy of Sciences in Wuhan (Gao et al., 2009). Fresh young leaves from well‐grown plants were collected, wrapped in tin paper, flash‐frozen in liquid nitrogen, and then stored at −80°C before use.

### 2.2 DNA extraction and sequencing

A plant genomic DNA extraction kit (TIANGEN) was used to extract total DNA from the samples. After the quality of the total DNA samples was confirmed by Shanghai Hanyu Biotechnology Co., Ltd., the samples were subjected to bidirectional sequencing using an Illumina HiSeq 2500, and the raw data obtained were converted into raw reads by CASAVA base‐calling analysis. The clean data obtained after removing the adaptor‐containing, low‐quality sequences were taken for subsequent analysis. Data processing was performed by Trimmomatic v0.32 (Bolger et al., 2014) with the following steps: (a) removal of sequences containing N bases; (b) removal of adaptor sequences in the reads; (c) removal of low‐quality bases (*Q* value < 20) from the reads in the 3′ to 5′ direction; (d) removal of low‐quality bases (*Q* value < 20) from the reads in the 5′ to 3′ direction; (e) removal of four bases with an average base quality <20; and (f) removal of the reads and their pairs with a length <50 nt. Velvet v1.2.03 (Zerbino & Birney, 2008) was used to assemble the clean data.

### 2.3 Characterization of chloroplast genomes

The cp genome of *Alsophila spinulosa* was used as the reference genome, and Dual Organellar GenoMe Annotator (DOGMA) (Milne et al., 2010) was used to predict the protein‐coding genes, rRNA genes, and tRNA genes in other genomes. Geneious Prime (Kearse et al., 2012) was used for manual correction according to the reference genome. The Shuffle‐Lagan mode in the online software mVISTA (Frazer et al., 2004) was used for genome‐wide comparison. Organellar Genome DRAW (OGDRAW) (Lohse et al., 2007) was used to draw physical cp genome maps, and Sequin software was used for submission of the cp genome of *G. denticulata*. Microsatellite repeats were predicted using the software MISA (Beier et al., 2017). The threshold repeat number of mononucleotide units was set to 10, the threshold repeat number of dinucleotide units was set to six, the threshold repeat number of trinucleotide units was set to five, and the threshold repeat number of tetra‐, penta‐, and hexanucleotide units was set to three. The minimum distance between two SSRs was set to 0 bp, that is, there was no statistical compound SSR. The distribution characteristics of SSRs of different species in the whole genome and its different regions were compared and analyzed. Among these characteristics, the relative abundance refers to the number of SSRs in the unit sequence length (kb), and the relative density refers to the length of the SSRs (bp) in the unit sequence length (kb).

### 2.4 Phylogenetic analysis

The maximum likelihood (ML), Bayesian inference (BI), maximum parsimony (MP), and neighbor‐joining (NJ) methods were used for phylogenetic analysis. MAFFT software (Katoh & Standley, 2013) was used to align the complete cp genome sequences of eight species of Cyatheaceae and one species of *Cibotium*, *Cibotium barometz* (Linn.) J. Sm. A phylogenetic tree was constructed using *C. barometz* (Linn.) J. Sm. as an outgroup. When the ML, MP, and BI trees had been constructed, the whole cp genome was screened in MrModeltest software to obtain the optimal nucleotide substitution model (GTR + I + G) selected based on the Akaike information criterion, and the relevant parameters were estimated. The ML tree was constructed by the software RAxML8.0.20 (Stamatakis, 2014), GTRGAMMAI was selected as the nucleotide substitution model, and the confidence of the branch was completed using the bootstrap analysis in autoMR. The BI tree was constructed by MrBayes v3.2.0 software (Ronquist et al., 2012) and was estimated by running 2,000,000 generations (Nst = 6, rates = invgamma). The MP tree was constructed in PAUP 4.0 software (Swofford, 2002) with the bootstrap value set to 1,000. The NJ tree was constructed in MEGA 7.0 software (Kumar et al., 2016), and the maximum composite likelihood algorithm was selected with the bootstrap value set to 1,000 times. The resulting phylogenetic tree was viewed and edited in Figtree v 1.4.3 software.

### 2.5 Statistical analysis

When *Gymnosphaera* is considered an independent taxonomic unit at the genus level, the eight Cyatheaceae species are divided into three genera; that is, *G*. denticulata, *G*. *podophylla*, *G*. *metteniana*, and *G*. *gigantea* belong to the genus *Gymnosphaera*; *A*. *spinulosa* and *A*. *costularis* belong to the genus *Alsophila*; and *S*. *brunoniana* and *S*. *lepifera* belong to the genus *Sphaeropteris*. When *Gymnosphaera* is classified into the genus *Alsophila*, Cyatheaceae is divided into two genera. The Kruskal–Wallis *H* test and Mann–Whitney *U* test in IBM SPSS v22.0 software (Allen et al., 2014) were used to analyze the significance of differences between taxa when three genera and two genera were assumed, respectively. The statistical results covered the whole cp genome, the SSRs of different unit lengths in the cp genome, and the number, relative abundance, relative density, and GC content of SSRs and SSRs of different unit lengths in the intergenic spacer (IGS), large single‐copy (LSC), intronic, and coding sequence (CDS) regions of the cp genomes of the eight Cyatheaceae species. Photovoltaic (PV) cluster analysis using the ward linkage method in R v3.5.1 (R Core Team, 2013) was performed on the SSRs of each cp genome and its IGS and LSC regions and on the number, relative abundance, relative density, and GC content of mononucleotide SSRs of the cp genomes with the Euclidean distance as the measurement. The number of repetitions was 10,000.

## 3 RESULTS

### 3.1 Genome structures and characteristics

**FIGURE 1:**
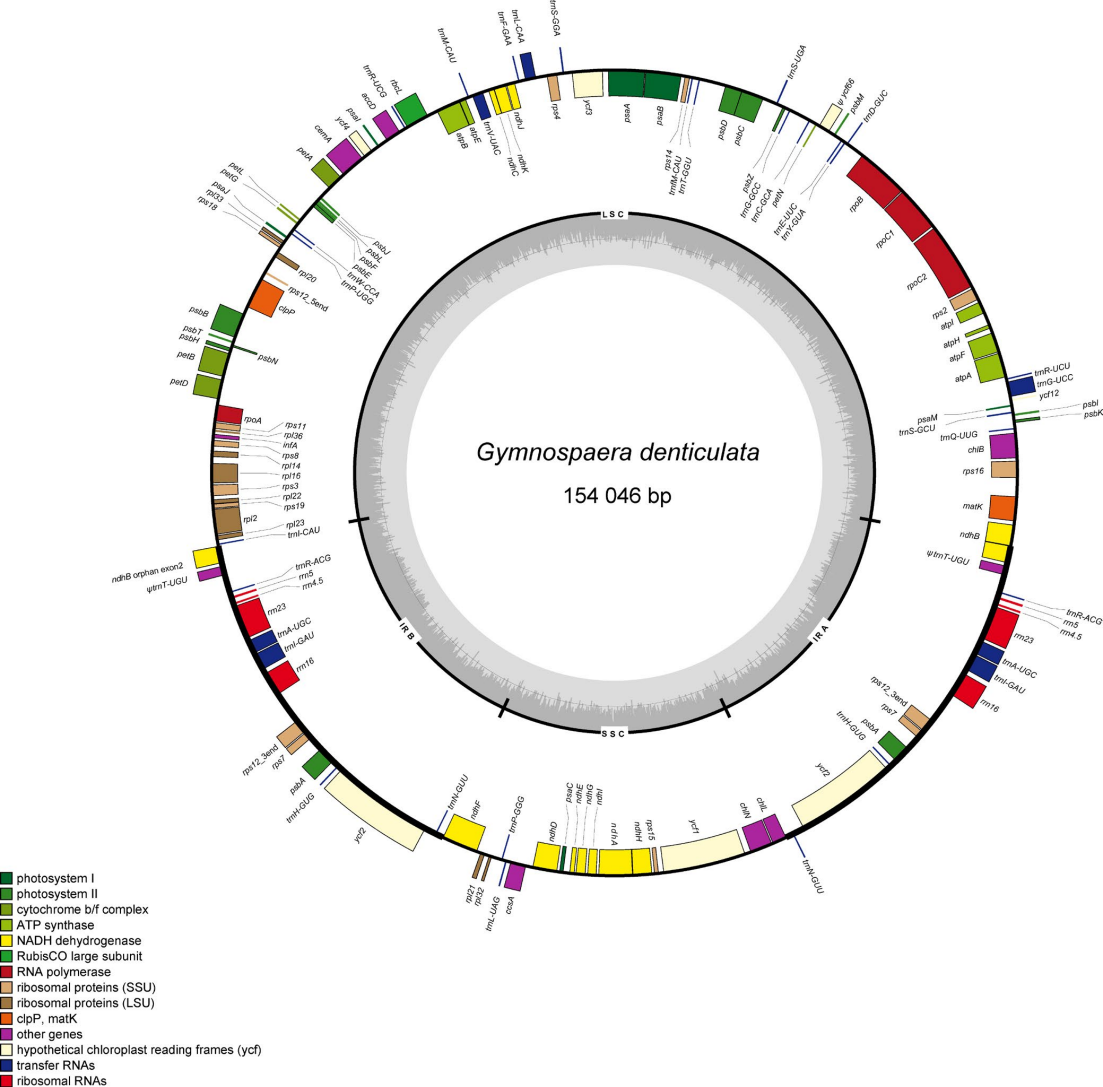
Gene map of the cp genome of *Gymnospaera denticulata*. Genes located in the outside of the outer circle are transcribed in the counterclockwise direction, whereas those in the inside of the circle are transcribed in the clockwise direction. Color codes represent different functional gene groups. In the middle circle, the GC and AT content variations are indicated by darker and lighter gray, respectively

The cp genomes of all eight Cyatheaceae species are double‐stranded, closed, circular molecules with a typical tetrad structure (with *G*. *denticulata* as an example, as shown in Figure 1). The genome length ranges from 154,046 bp (*A*. *denticulata*) to 166,151 bp (*A*. *gigantea*). The structure includes a large single‐copy region (LSC) (85,975–92,315 bp), a small single‐copy region (SSC) (23,245–28,874 bp), and an inverted repeat region (IR) (23,245–28,874 bp), where IRA and IRB are two inverted repeat regions. The GC content of each region of the cp genomes of different species varied little, with the total GC content ranging from 40.3% to 41.9% (Table 1). Only LSC, SSC, and one IR were analyzed. The cp genome of each Cyatheaceae species contained 117 genes, which encoded 85 proteins, four rRNAs, and 28 tRNAs. Pseudogenes (*ycf66, trnT‐UGU*) are also present in these genomes. Among these genes, 13 are located in the IR region. The *ndhB* gene spans the LSC and IRA regions, and there is a duplicated exon 2 sequence of the *ndhB* gene is present near the boundary of the IRB. Twelve genes have one intron, and three genes (*ycf3*, *clpP*, and *rps12*) have two introns.

**TABLE 1:** List of the eight Cyatheaceae species, GenBank accession numbers, and structural features of the chloroplast genomes

Species	GenBank No.	LSC			IR			SSC			Total	
		Length (bp)	GC%	Length (%)	Length (bp)	GC%	Length (%)	Length (bp)	GC%	Length (%)	Length (bp)	GC%
*G. denticulata*	MT726940	85,975	40.0	55.8	23,245	40.8	15.1	21,581	38.1	14.0	154,046	40.6
*G. podophylla*	MG262389	86,762	40.0	52.2	28,874	46.2	17.4	21,641	38.1	13.0	166,151	41.9
*G. gigantea*	MH603068	92,315	41.1	57.1	23,831	43.3	14.7	21,702	38.2	13.4	161,679	41.3
*G. metteniana*	MN795320	92,292	41.1	57.1	23,822	43.3	14.7	21,666	38.1	13.4	161,602	41.3
*A. costularis*	MH684489	86,338	39.6	55.1	24,356	43.0	15.5	21,625	37.8	13.8	156,675	40.5
*A. spinulosa*	NC_012818	86,308	39.6	55.1	24,365	43.0	15.6	21,623	37.9	13.8	156,661	40.4
*S. brunoniana*	MT543220	86,196	39.2	55.0	24,011	43.2	15.3	22,441	38.1	14.3	156,659	40.3
*S. lepifera*	MN623357	86,349	39.3	53.3	24,067	43.2	14.8	27,733	41.5	17.1	162,216	40.8

Abbreviations: IR, inverted repeat region; LSC, Large single copy region; SSC, small single copy region.

### 3.2 Analysis of the characteristics of SSRs

**FIGURE 2:**
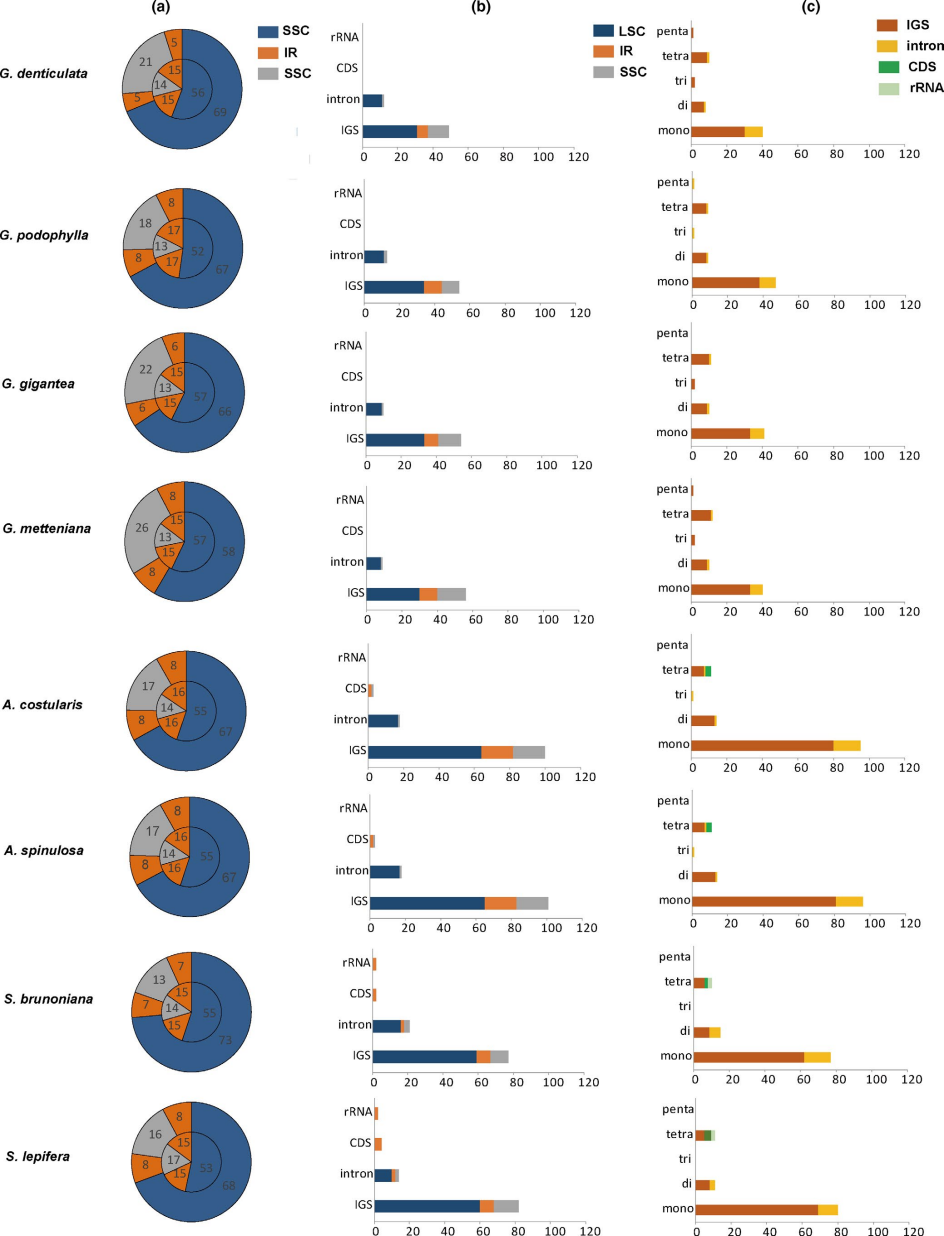
Comparison of microsatellite repeats among the eight Cyatheaceae chloroplast genomes. (a) The inner circle is the distribution ratio of the four regions (LSC, SSC, and IRs) in the genome, and the outer circle is the distribution ratio of SSRs between the four regions. (b) Distribution ratio of SSRs in different regions (LSC, SSC, and IRs) of chloroplast genome. (c) Ratio of mono‐ to pentanucleotide SSRs in different regions (IGS, intron, CDS, and rRNA gene) of the chloroplast genome. Numbers represent the distribution ratio of SSR numbers. LSC: large single copy region; SSC: small single copy region; IR: inverted repeat region; IGS: intergenic spacer region; CDS: coding sequence region

The number, relative abundance, relative density, and GC content of SSRs in the cp genomes of all eight Cyatheaceae species were systematically compared (Table 2). The number (121–122), relative abundance (0.77–0.78/bp), relative density (9.81–9.82 bp/kb), and GC content (0.18–0.20) of SSRs in the cp genomes of *A*. *spinulosa* and *A*. *costularis*; the number (102), relative abundance (0.63–0.65/bp), relative density (6.70–8.18 bp/kb), and GC content (0.08–0.10) of SSRs in the cp genomes of *S*. *brunoniana* and *S*. *lepifera*; and the number (61–67), relative abundance (0.40/bp), relative density (4.11–5.06 bp/kb), and GC content (0.22–0.29) of SSRs of *G. denticulata*, *G. podophylla*, *G. metteniana*, and *G. gigantea* had similar values, which were not proportional to the sizes of the genomes. When Gymnosphaera was considered as an independent classification unit at the genus level, the eight species of Cyatheaceae were divided into three genera. That is, *G*. *denticulata*, *G*. *podophylla*, *G*. *metteniana*, and *G*. *gigantea* belonged to the genus *Gymnosphaera*, *A. spinulosa* and *A. costularis* belonged to the genus *Alsophila*, and *S. brunoniana* and *S. lepifera* belonged to the genus *Sphaeropteris*, which indicated that in the phylogenetic context of the three genera, the characteristics of SSRs are genus specific at the level of the genome. In *Alsophila*, the number, relative abundance, and relative density of SSRs were the highest among the eight species, and they were the smallest in *Gymnosphaera*. The highest GC content was found in *Gymnosphaera*, and the lowest in *Sphaeropteris*. The proportions of GC bases in the cp genomes of the eight species of Cyatheaceae were much lower than the proportions of AT. The proportion of SSRs in the IR region was 2–3.3 times the proportion of IR sequences among the whole genome sequence (Figure 2a).

**TABLE 2:** Overview of the eight Cyatheaceae chloroplast genomes and characteristics of their SSRs

	*G. denticulata*	*G. podophylla*	*G. gigantea*	*G. metteniana*	*A. costularis*	*A. spinulosa*	*S. brunoniana*	*S. lepifera*
Sequence analyzed (kb)	154.05	166.15	161.68	161.60	156.68	156.66	156.66	162.22
No. of SSRs	61	67	64	65	121	122	102	103
Relative abundance (No./kb)	0.40	0.40	0.40	0.40	0.77	0.78	0.65	0.63
Total length of SSRs (bp)	634	802	818	801	1539	1538	1,352	1,327
Relative density (bp/kb)	4.11	4.82	5.06	4.96	9.82	9.81	8.63	8.18
GC content	0.30	0.25	0.29	0.22	0.20	0.18	0.11	0.10
Genome content	0.004	0.005	0.005	0.005	0.010	0.010	0.007	0.008

The number, relative abundance, relative density, and GC content of SSRs in the cp genomes of the three Cyatheaceae genera also had similar values in different regions of the genome (LSC, SSC, and IR; IGS, intron, CDS, and rRNA gene regions), indicating that in the phylogenetic context of the three genera, SSR characteristics were genus specific at the level of different regions of the genome (Figure 2; Appendix Tables S9 and S10). For the distributions of SSRs on the genome, the SSRs located in LSC (58.46%–73.52%) were most enriched in each species, followed by SSC (12.65%–21.08%), and the least in IR (9.84%–16.53%). The number of SSRs was highest in LSC and IR in *Alsophila* (81–82, 20) and lowest in *Gymnosphaera* (42–45, 8–10). Furthermore, SSRs accounted for 75.5%–86.2%, 13.7%–20.6%, 2.0%–3.9%, and 2.0% of the IGS, intron, CDS, and rRNA gene regions (pseudogenes were treated as IGS regions). Among them, SSRs were detected only in the CDS regions of the cp genomes of *Sphaeropteris* and *Alsophila*, and SSRs were detected in the rRNA genes of the cp genomes of *Sphaeropteris*. The SSRs at IGS regions were most enriched in *Alsophila* (100–101) and the least in *Gymnosphaera* (49–56). The number of SSRs located in the intron regions was 18 in *Alsophila* and 9–13 in *Gymnosphaera*. These results showed that in the phylogenetic context dividing the eight Cyatheaceae species into three genera, different taxa had different patterns of SSR characteristics in the cp genome and its different regions, namely, the SSR characteristics of the cp genomes of the eight Cyatheaceae species were consistent with their phylogenetic relationship.

### 3.3 Analysis of the types and characteristics of SSRs of different nucleotide numbers

The proportions of mono‐, di‐, tri‐, tetra‐, and pentanucleotide SSRs in each species were 62.5%–78.0%, 10.6%–15.6%, 0%–3.3%, 9.0%–18.5%, and 0%–1.5%, respectively. In the distribution of species, the number of mononucleotide had obvious genus specificity: *Gymnosphaera* (40–47), *Sphaeropteris* (77–80), and *Alsophila* (95–96). No hexanucleotide SSRs were detected. Among mononucleotide repeats, more A/T motifs were observed, and the dinucleotide repeats were dominated by AT/TA motifs. There were more tetranucleotide SSRs than tri‐, and pentanucleotide SSRs. Trinucleotide SSRs do not exist in *Sphaeropteris*, and pentanucleotide SSRs do not exist in *Sphaeropteris*, *Alsophila,* and *G*. *gigantea*. The mono‐, di‐, tri‐, tetra‐, and pentanucleotide SSRs of the cp genomes of the three genera were present in similar numbers, relative abundance, relative density, and GC content at the level of the genome and in the specific regions of the genome (LSC, SSC, and IRs; IGS, intron, CDS, and rRNA gene regions), which was especially true for mononucleotide and dinucleotide SSRs (Table 3; Appendix Tables S9 and S10).

**TABLE 3:** The number, relative abundance, relative density, and GC content of mono‐ to pentanucleotide SSRs in the eight chloroplast genomes of Cyatheaceae

Repeat type	Characteristics of SSR	*G. denticulata*	*G. podophylla*	*G. gigantea*	*G. metteniana*	*A. costularis*	*A. spinulosa*	*S. brunoniana*	*S. lepifera*
Mononucleotide	No. of SSRs	40	47	41	40	95	96	77	80
	Abundance (No./kb)	0.26	0.28	0.25	0.25	0.61	0.61	0.49	0.49
	Density (bp/kb)	2.93	3.16	3.13	2.88	7.67	7.60	6.41	6.23
	GC	0.36	0.35	0.40	0.3	0.22	0.19	0.08	0.09
Dinucleotide	No. of SSRs	8	9	10	10	14	14	15	11
	Abundance (No./kb)	0.05	0.05	0.06	0.06	0.09	0.09	0.10	0.07
	Density (bp/kb)	0.69	0.83	0.94	0.92	1.35	1.35	1.40	1.04
	GC	0.06	0	0.08	0.05	0	0	0.23	0.12
Trinucleotide	No. of SSRs	2	1	2	2	1	1	0	0
	Abundance (No./kb)	0.01	0.01	0.01	0.01	0.01	0.01	0	0
	Density (bp/kb)	0.16	0.07	0.15	0.15	0.08	0.08	0	0
	GC	0	0	0	0	0.33	0.33	0	0
Tetranucleotide	No. of SSRs	10	9	11	12	11	11	10	12
	Abundance (No./kb)	0.06	0.05	0.07	0.07	0.07	0.07	0.06	0.07
	Density (bp/kb)	0.80	0.67	0.84	0.92	0.87	0.87	0.82	0.91
	GC	0.18	0.14	0.18	0.16	0.29	0.29	0.3	0.21
Pentanucleotide	No. of SSRs	1	1	0	1	0	0	0	0
	Abundance (No./kb)	0.01	0.01	0	0.01	0	0	0	0
	Density (bp/kb)	0.10	0.09	0	0.09	0	0	0	0
	GC	0	0.4	0	0.4	0	0	0	0

**FIGURE 3:**
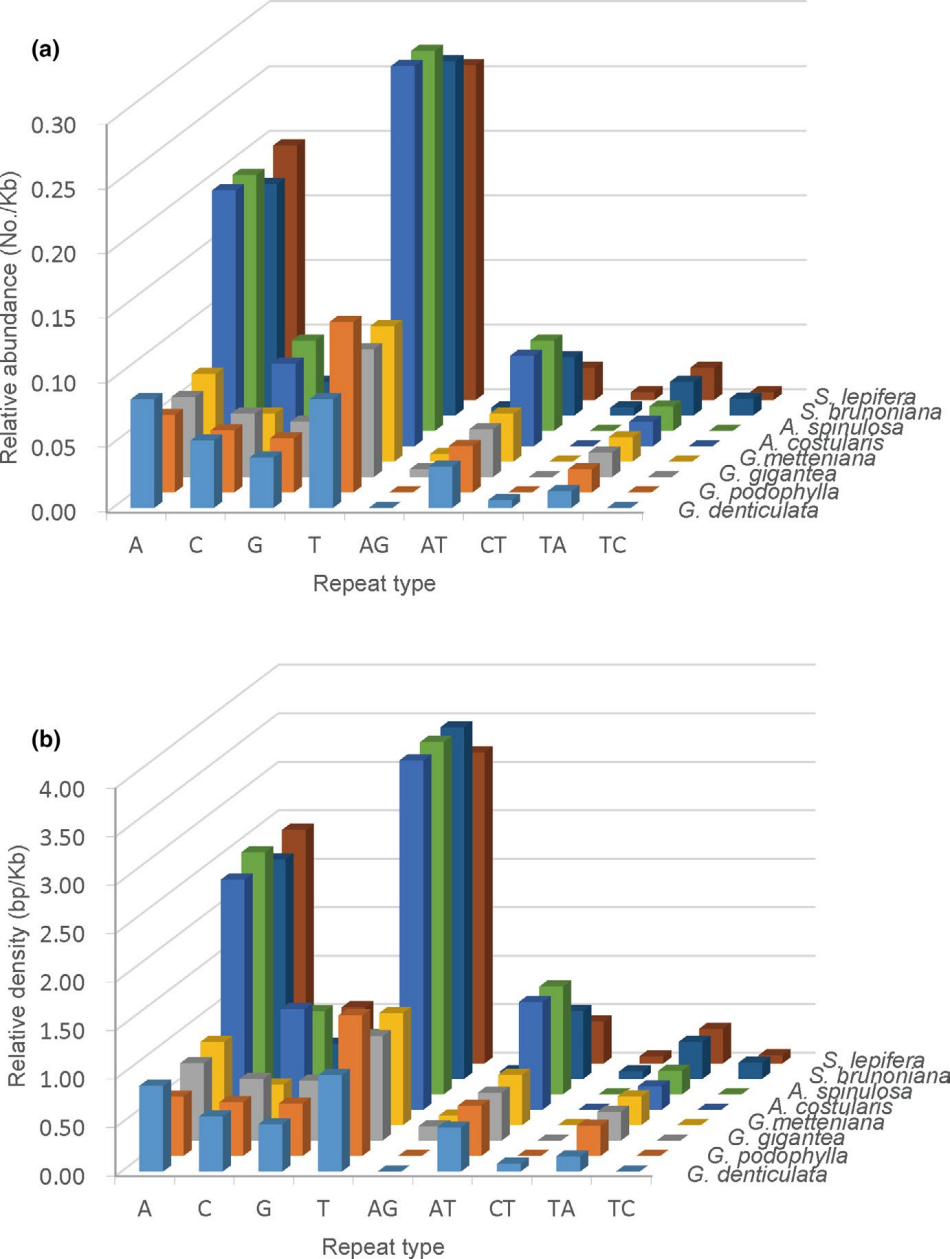
Relative abundance and relative density of mono‐ and dinucleotide SSRs in the eight chloroplast genomes of Cyatheaceae

The number, relative abundance, relative density, and GC content of SSRs of different unit lengths in the cp genome and its different regions had genus specificity in the phylogenetic context of dividing the eight Cyatheaceae species into three genera. In addition, the number, relative abundance, and relative density of SSRs of different base types in the cp genomes of the three genera of plants also had genus specificity, which was especially true for mono‐ and dinucleotide SSRs (Figure 3; Appendix Table S9). *Alsophila* had the highest A/T and C/G motif content (77, 18–19), *Gymnosphaera* had the least A/T motif content (26–32), and *Sphaeropteris* had the least C/G motif content (6).

### 3.4 Phylogenetic analysis

**FIGURE 4:**
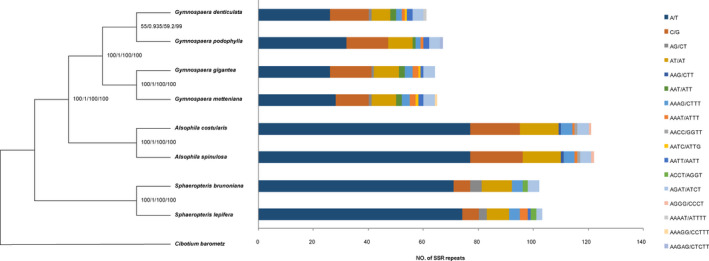
Ml, BI, MP, and NJ phylogenetic trees based on eight complete chloroplast genome sequences in Cyatheaceae and the distribution of different types of SSR motifs. The outgroup is *Cibotium barometz*

The cp genomes of the eight species of Cyatheaceae were compared globally, and the phylogenetic trees were constructed with four methods (ML, BI, MP, and NJ) using *C*. *barometz* (Linn.) J. Sm. as an outgroup, as shown in Figure 4. The topologies of the four trees were consistent, except that the support rate of the branches of the *G. denticulata* and *G. gigantea* was lower (the bootstrap values with the ML, MP, and NJ methods were 55%, 59.2%, and 99%, respectively, and the posterior probability of BI was 0.935). The support rate of the other branches was higher (the bootstrap values with the ML, MP, and NJ methods were all 100%, and the posterior probability of BI was 1.00). Closely related *S*. *brunoniana* and *S*. *lepifera* were clustered into one branch, which was located at the base of the phylogenetic tree, indicating that this group diverged earlier in this family. *G. denticulata*, *G. podophylla*, *G. gigantea*, and *G. metteniana* were clustered into one branch, which was located inside the branch of *S*. *brunoniana* and *S*. *lepifera* and was a sister group of the branch formed by *A*. *spinulosa* and *A*. *costularis*.

### 3.5 Statistical analysis of the results

**FIGURE 5:**
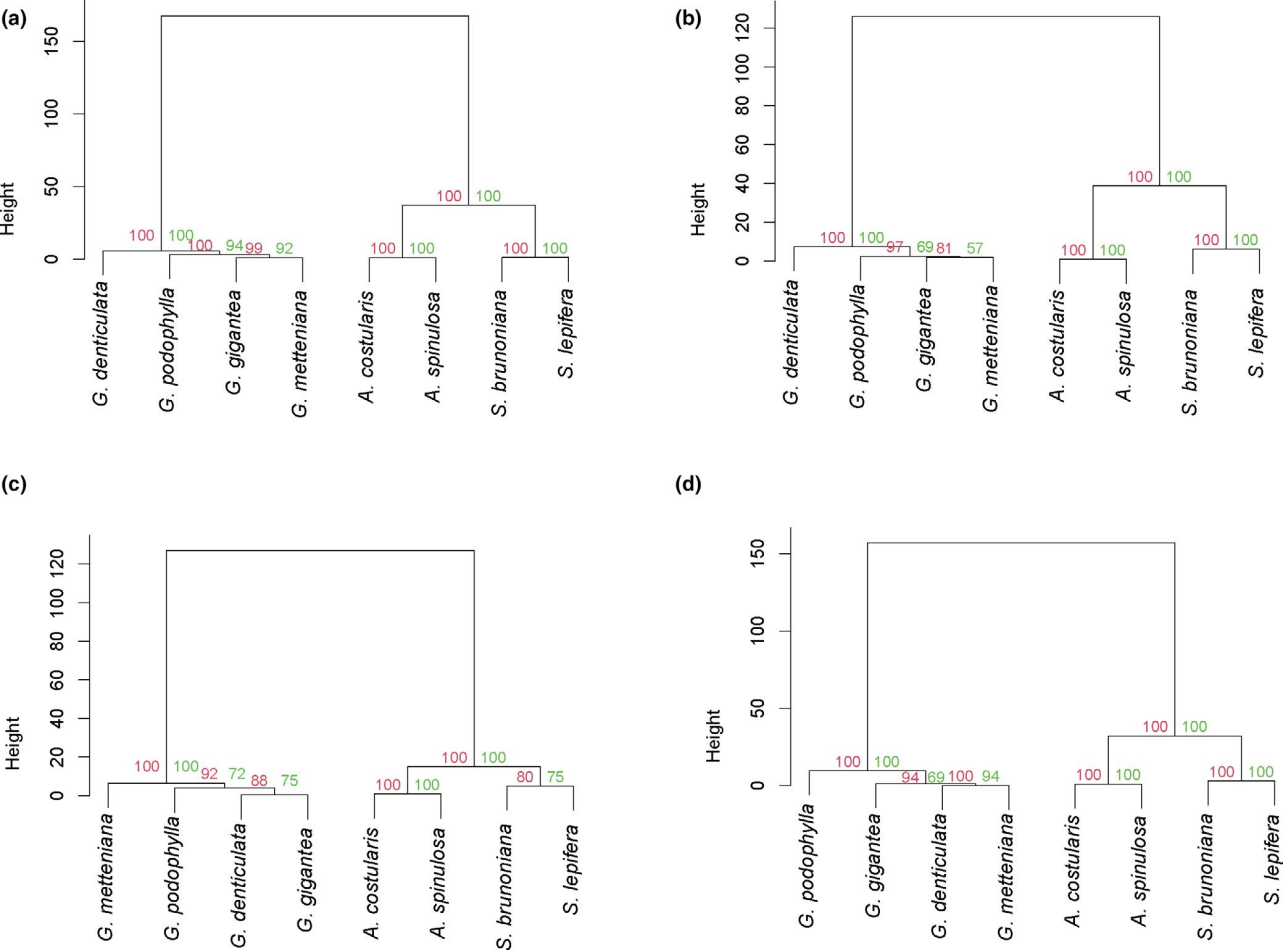
Clustering analysis of eight Cyatheaceae species based on the number, relative abundance, relative density, and GC content of SSRs across the whole chloroplast genome (a), IGS (b), LSC (c), and mononucleotide (d) SSRs in the chloroplast genomes

The Kruskal–Wallis H test and Mann–Whitney *U* test values are shown in Appendix Table S11. In this study, only SSRs in the IGS and LSC regions of the cp genome, as well as mononucleotide SSRs in the whole cp genomes, were considered. Significant differences in the number, relative abundance, relative density, and GC content of SSRs were observed when the eight Cyatheaceae species were divided into three genera (Kruskal–Wallis *H*, *p* < .05). When *Gymnosphaera* was included in the genus *Alsophila*, two genera were defined, and only the difference in the GC content was significant (Table 4). The number of SSRs in other regions of the cp genome and the number of SSRs of other unit lengths were small, so they are not discussed in this study. The clustering results for the number, relative abundance, relative density, and GC content of the SSRs in the cp genomes and their IGS regions and the mononucleotide SSRs of the whole cp genomes of the eight Cyatheaceae species (Figure 5) showed that the eight species were divided into two groups. That is, *S*. *brunoniana*, *S*. *lepifera*, *A*. *spinulosa,* and *A*. *costularis* were in one group, and *G. denticulata*, *G. podophylla*, *G. metteniana*, and *G. gigantea* were in the other group.

**TABLE 4:** Significance test of the number, relative abundance, relative density, and GC content of SSRs in the whole cp genome, IGS, and LSC and mononucleotide SSRs in the whole cp genomes of the eight Cyatheaceae species

Classification treatment	Genus	Species	SSR Characteristics	*P*				Literature cited
				Genome	IGS	LSC	Mononucleotide	
Three genera	*Gymnosphaera*	*G. denticulata* *G. podophylla*	No. of SSRs	0.050*	0.048*	0.048*	0.050*	Smith et al. (2006); Korall et al. (2007); Janssen and Rakotondrainibe (2008); Korall and Pryer (2014); Ching (1978); Dong and Zuo (2018)
		*G. gigantea* *G. metteniana*	Relative abundance (No./kb)	0.033*	0.050*	0.050*	0.050*	
	*Alsophila*	*A. costularis* *A. spinulosa*	Relative density (bp/kb)	0.050*	0.050*	0.050*	0.050*	
	*Sphaeropteris*	*S. brunoniana* *S. lepifera*	GC content	0.050*	0.050*	0.050*	0.050*	
Two genera	*Alsophila*	*G. G. denticulata* *G. podophylla*	No. of SSRs	0.502	0.502	0.502	0.502	PPG I (2016); Xia (1989); Zhang and Nishida (2013)
		*G. gigantea* *G. metteniana*	Relative abundance (No./kb)	0.478	0.505	0.505	0.505	
		*A. costularis* *A. spinulosa*	Relative density (bp/kb)	0.505	0.505	0.505	0.505	
	*Sphaeropteris*	*S. brunoniana* *S. lepifera*	GC content	0.046*	0.046*	0.046*	0.046*	

Note

When Cyatheaceae plants are treated as three genera, the Kruskal–Wallis *H* test is used; when treated as two genera, the Mann–Whitney *U* test is used; *p ≤ .05.

Abbreviations: IGS, intergenic spacer region; LSC, large single copy region.

## 4 DISCUSSION

### 4.1 Characteristics of the cpSSRs of the eight Cyatheaceae species

The cp plastomes of the eight Cyatheaceae species are very conservative and are similar in structure and gene content (117 genes). The types and order of genes are the same. The lower distribution of SSRs (Figure 2a) in the IR region may be related to the higher mismatch repair rate and lower mutation rate in the IR region (Ellegren, 2004; Li et al., 2016). The lower GC content (Tables 2 and 3) of SSRs may be associated with the tendency of GC‐rich regions toward AT mutations (Kuang et al., 2011; Ren et al., 2007). SSRs are mainly located in intergenic and noncoding regions, with a few present in exons (Li et al., 2004; Su et al., 2018), and the results of this study are consistent with this information. This phenomenon is related to negative selection against frameshift mutations in coding regions (Metzgar et al., 2000). CpSSRs are characterized by high variability and codominant inheritance (Chmielewski et al., 2015; George et al., 2015); therefore, their sequences can be used to effectively classify low taxonomic and closely related groups and variant plant subspecies.

In this study, we used MISA to scan the recently assembled Cyatheaceae cp genomes for microsatellites of 1–6 bp. The cp genomes of eight Cyatheaceae species were similarly analyzed using the same bioinformatics tool and search parameters to compare our results. The number, relative abundance, relative density, and GC content of the cpSSRs of *A*. *spinulosa* and *A*. *costularis*; the number, relative abundance, relative density, and GC content of the cpSSRs of *S*. *brunoniana* and *S*. *lepifera*; and the number, relative abundance, relative density, and GC content of the cpSSRs of *G. denticulata*, *G. podophylla*, *G. metteniana*, and *G. gigantea* had similar values. Based on these findings, the eight Cyatheaceae species were divided into three groups, which is consistent with the recent studies (Ching, 1978; Dong & Zuo, 2018; Janssen & Rakotondrainibe, 2008; Korall et al., 2007; Korall & Pryer, 2014; Smith et al., 2006), in which eight Cyatheaceae species were divided into three genera, indicating that the characteristics of the cpSSRs showed genus specificity at the genome level in the phylogenetic context of the three genera (Figure 2, Tables 2 and 3, and Appendix Tables S9–S11).

The number, relative abundance, and relative density of the cpSSRs of *Alsophila* were lower than those of *Gymnosphaera*, while those of *Sphaeropteris* fell between the two. The numbers of SSRs of *Alsophila*, *Gymnosphaera,* and *Sphaeropteris* distributed in the LSC region were 81–82 (66.94%–67.21%), 38–45 (58.46%–68.85%), and 70–75 (67.96%–73.52%), respectively, which was the most in each species, followed by the SSC region, and the least in the IR region (Appendix Table S10). The numbers of SSRs of *Alsophila*, *Gymnosphaera,* and *Sphaeropteris* distributed in the IGS region were 99–100 (81.81%–81.97%), 49–56 (80.32%–86.15%), and 77–83 (75.49%–80.58%), respectively, which was the most in each species, followed by the intron region. SSRs were detected in the CDS region of *Alsophila* and *Sphaeropteris*, and SSRs were detected only in the rRNA gene of *Sphaeropteris* (Appendix Table S10). The cpSSRs of each species were mainly distributed in the IGS region and LSC regions, which is consistent with that reported in *Adrinandra* (Nguyen et al., 2021), *Blumea* (Abdullah et al., 2021), *Mikania* (Su et al., 2018), *Prunus* (Huang et al., 2021), Cupressaceae (Ping et al., 2021), Poaceae (Wei et al., 2021), Polypodiaceae (Liu et al., 2021), and ferns (Fan et al., 2021). *Alsophila* had the highest relative abundance and density (0.77–0.78/kb, 9.81–9.82 bp/kb, respectively) of the SSRs in the cp genome, and the lowest relative abundance and density (0.40/kb, 4.11–5.06 bp/kb) were found in *Gymnosphaera*. The number, relative abundances, and density of SSRs showed great similarity across eukaryotic genomes among taxonomic groups (Manee et al., 2020; Qi et al., 2015; Srivastava et al., 2019).

The number, relative abundance, relative density, and GC content of SSRs of different unit length in Cyatheaceae cp genomes were also genus specific (Table 3; Appendix Tables S9–S11), which was especially true for mononucleotide and dinucleotide SSRs (Figure 3), possibly because of the lower content of SSRs of other unit lengths. The number of mononucleotide repeats of *Alsophila*, *Gymnosphaera,* and *Sphaeropteris* was the largest at 77–80 (75.49%–77.67%), 40–47 (61.54%–70.15%), and 95–96 (78.51%–78.69%), and the proportions of the A/T repeat motif were 80.21%–81.05%, 63.41%–70%, and 92.21%–92.50%, respectively. Dinucleotide repeats of *Alsophila*, *Gymnosphaera,* and *Sphaeropteris* showed two types of repeat motifs (AG/CT, AT/AT), with numbers of 9–14, 8–14, and 11–15 and AT/AT accounting for 100%, 87.50%–100%, and 72.73%–73.33% of the motifs, respectively (Appendix Tables S9–S11). Mononucleotide SSRs which exist in a large numbers in cp genomes (George et al., 2015; Liang et al., 2019) are the most abundant (Table 3), and A/T motifs are the most common (Figure 3; Appendix Table S9). This finding is similar to previously reported patterns of land plants (George et al., 2015; Huang et al., 2021; Ren et al., 2021; Vieira et al., 2014; Wei et al., 2021). However, in Polypodiaceae plastomes, most repeats were C/G mononucleotides. This increase in GC content may be related to the adaptation mechanism of Polypodiaceae to the environment (Gao et al., 2018; Liu et al., 2021). These results indicate that SSRs in cp genomes can reflect genetic variation between different taxa.

The distribution of different repeat types (from mononucleotide to hexanucleotide) of motifs in coding and noncoding regions, introns, and intergenic regions displayed a high degree of genus specificity (Appendix Tables S10 and S11), which can be partially explained by the interaction of mutation mechanisms and differential selection (Toth et al., 2000). The most common mutation mechanism affecting SSRs is slipped replication. Other mechanisms, such as unequal crossing over, nucleotide substitution, and duplication events, are also responsible for SSR variation (Hancock, 1999; Schlotterer & Tautz, 1992). The SSRs of different groups of genomes have specific distribution patterns, which are related to their common ancestors. Evolutionary trends have been linked to the inclusion of SSRs, which may have been preserved because of their ability to adapt to novel regulatory mechanisms (Srivastava et al., 2019). Analysis of the characteristics of SSRs provides useful clues for the phylogenetic study of Cyatheaceae and facilitates an understanding of the evolution of SSRs in plant genomes.

### 4.2 Phylogenetic significance of SSR characteristics of the cp genomes of the eight Cyatheaceae species

Dong and Zuo (2018) pointed out that *Gymnosphaera* and *Alsophila* were significantly different in morphological traits such as petiole color, the presence or absence of degenerated pinnae at the base, and the presence or absence of indusium and sporogenesis, and advocated for the restoration of the hierarchical status of the genus *Gymnosphaera* to reflect the divergence mechanisms of this group of plants in terms of molecular phylogeny, morphology, and sporogenesis. Based on five chloroplast DNA regions (*rbcL*, *rbcL‐accD*, *rbcL‐atpB*, *trnG‐trnR*, and *trnL‐trnF*), the results of the phylogenetic analysis of Cyatheaceae conducted by Dong and Zuo (2018) support the independence of Gymnosphaera. Since the stipe of *Sphaeropteris* is stramineous or purple, not black, and the cells of the scale are all essentially similar to the edge of the scale, which is often toothed or even ciliate, *Sphaeropteris* can be clearly distinguished from other genera in the family Cyatheaceae. Molecular phylogenetic studies further confirm this point (Dong & Zuo, 2018; Janssen & Rakotondrainibe, 2008; Korall & Pryer, 2014; Wang et al., 2003).

In the recent studies, eight Cyatheaceae species were divided into three genera (Ching, 1978; Dong & Zuo, 2018; Janssen & Rakotondrainibe, 2008; Korall et al., 2007; Korall & Pryer, 2014; Smith et al., 2006) or two genera (PPG I, 2016; Xia, 1989; Zhang & Nishida, 2013), in which case SSRs were compared by the Kruskal–Wallis *H* test or the Mann–Whitney *U* test, respectively, and the results showed that the statistical and clustering results showed that their distribution was consistent with the recent classification of Cyatheaceae which divided the eight Cyatheaceae species into three genera, which was confirmed by the PV clustering analysis of SSRs. In this study, phylogenetic trees were constructed using the cp genomes of eight Cyatheaceae species, which showed that *G*. *denticulata*, *G*. *podophylla*, *G*. *gigantea*, *G*. *metteniana*, *A. costularis*, and *A. spinulosa* formed a monophyletic clade. In general, phylogenetic relationships from sequence construction are not sufficient to indicate whether two taxa are distinct. The phylogenetic relationships from SSR characteristics cluster analysis can distinguish the two groups. The different clustering results may be related to the use of different clustering methods and different data. In this phylogenetic context of the three genera, the SSR characteristics have genus specificity, which may reflect a universal law among Cyatheaceae genera.

Recent studies have shown minimal differences in the distribution patterns and numbers of SSRs among cpSSRs of the related species (Fan et al., 2021; Liu et al., 2021; Ping et al., 2021; Wei et al., 2021). Comparative analysis of cpSSRs from broad plant groups may be useful for a better understanding of the diversity and evolutionary trends of cp genomes (George et al., 2015). This study indicates that the distribution characteristics of cpSSRs of Cyatheaceae can provide useful phylogeny information at the genus level. Relatively few studies have explored phylogenetic relationships in ferns by analyzing cpSSRs. Since the software programs that identify SSRs are limited by their efficiency and parameter settings and may also be affected by the quality of the SSR dataset generated, their accuracy requires improvement (Ellegren, 2004; Lim et al., 2013). Given the limitations of the current plant genome sequences, we did not analyze the large‐scale SSR characteristics of the cp genomes of Cyatheaceae, nor did we obtain the plant materials of *Cyathea* that are generally distributed in South America. However, this study is based on the existing chloroplast genomes of Cyatheaceae and the eight species encompass most Cyatheaceae genera. Our results demonstrate that the distribution characteristics of the cpSSRs of the existing Cyatheaceae are genus specific. Our aim was not to solve the phylogenetic problem of Cyatheaceae but to identify traits that can be used for phylogenetics. This study provides a new basis for the classification of Cyatheaceae at the species and genus levels, thus advancing the phylogenetic study of Cyatheaceae. In the future, more genomic and transcriptomic data are needed to validate these results.

## 5 CONCLUSION

The cp genomes of the eight Cyatheaceae species have the same gene types in the same order and similar structure and gene content. The distribution characteristics of the cpSSRs of the eight species are consistent with the recent classification of Cyatheaceae, which divides the eight Cyatheaceae species into three genera, indicating that in the phylogenetic context of the three genera, the distribution characteristics of SSRs in their cp genomes are genus specific, which may be a general rule among Cyatheaceae. Analyzing the characteristics of SSRs provides clues and new ideas for research on the phylogeny of Cyatheaceae.

## CONFLICT OF INTEREST

The authors declare no competing interests.

## AUTHOR CONTRIBUTION

**Ming Zhu:** Conceptualization (equal); Data curation (lead); Formal analysis (lead); Writing‐original draft (lead). **Peipei Feng:** Data curation (equal); Writing‐original draft (equal). **Jingyao Ping:** Data curation (equal); Writing‐original draft (equal). **Jinye Li:** Data curation (supporting); Writing‐original draft (supporting). **YingJuan Su:** Conceptualization (lead); Writing‐original draft (equal). **Ting Wang:** Conceptualization (lead); Writing‐original draft (equal).

## Supporting information

AppendixTable S1‐S11

## Data Availability

The chloroplast genomes of *Gymnosphaera denticulata* Baker and *Gymnosphaera metteniana* Hance were deposited in GenBank (https://www.ncbi.nlm.nih.gov/genbank/) and under the accession numbers of MT726940 and MN795320, respectively.
