# Supplementary material for: Phylogenetic significance of the characteristics of simple sequence repeats at the genus level based on the complete chloroplast genome sequences of Cyatheaceae
Source: Ecol Evol. 2021 Sep 23;11(20):14327–40. doi: 10.1002/ece3.8151 (PMC8525152; doi:10.1002/ece3.8151)
Supplement: Supplementary file 1 — AppendixTable S1‐S11 [file ECE3-11-14327-s001.docx]

Appendix Table S1 List of simple sequence repeats (SSRs) in the chloroplast genomes of *Gymnospaera denticulata*

| SSR nr. | SSR type | Locus | Region | SSR motif | size | star | end | Location |
| --- | --- | --- | --- | --- | --- | --- | --- | --- |
| 1 | p5 | spacer | LSC | ATTTT | 15 | 5331 | 5345 | *rps16-chlB* |
| 2 | p1 | spacer | LSC | G | 14 | 7040 | 7053 | *chlB-trnQ(UGG)* |
| 3 | p1 | spacer | LSC | C | 11 | 8373 | 8383 | *trnS(GCU)-psaM* |
| 4 | p1 | intron | LSC | A | 12 | 9401 | 9412 | *trnG(UCC)* |
| 5 | p4 | intron | LSC | AATT | 12 | 9648 | 9659 | *trnG(UCC)* |
| 6 | p1 | spacer | LSC | G | 16 | 13299 | 13314 | *atpF-atpH* |
| 7 | p1 | spacer | LSC | C | 12 | 14280 | 14291 | *atpH-atpI* |
| 8 | p1 | spacer | LSC | C | 12 | 15231 | 15242 | *atpI-rps2* |
| 9 | p1 | intron | LSC | C | 10 | 22475 | 22484 | *rpoC1* |
| 10 | p1 | spacer | LSC | A | 11 | 23468 | 23478 | *rpoC1-rpoB* |
| 11 | p1 | spacer | LSC | C | 10 | 26793 | 26802 | *rpoB-trnD(GUC)* |
| 12 | p4 | spacer | LSC | ATAG | 12 | 27725 | 27736 | *rpoB-trnD(GUC)* |
| 13 | p1 | spacer | LSC | T | 13 | 28419 | 28431 | *trnE(UUC)-psbM* |
| 14 | p4 | spacer | LSC | TGAT | 12 | 28511 | 28522 | *trnE(UUC)-psbM* |
| 15 | p1 | spacer | LSC | A | 10 | 29560 | 29569 | *ycf66* |
| 16 | p1 | spacer | LSC | G | 14 | 29640 | 29653 | *ycf66* |
| 17 | p1 | spacer | LSC | G | 10 | 31641 | 31650 | *trnC(GCA)-trnG(GCC)* |
| 18 | p1 | spacer | LSC | A | 10 | 31715 | 31724 | *trnC(GCA)-trnG(GCC)* |
| 19 | p1 | spacer | LSC | C | 10 | 32102 | 32111 | *trnG(GCC)-psbZ* |
| 20 | p4 | spacer | LSC | TATC | 16 | 33543 | 33558 | *trnS(UGA)-psbC* |
| 21 | p4 | spacer | LSC | ATAG | 12 | 33562 | 33573 | *trnS(UGA)-psbC* |
| 22 | p1 | spacer | LSC | A | 10 | 37593 | 37602 | *psbD-trnT(GGU)* |
| 23 | p1 | intron | LSC | A | 11 | 44104 | 44114 | *ycf3* |
| 24 | p1 | intron | LSC | T | 13 | 44123 | 44135 | *ycf3* |
| 25 | p1 | intron | LSC | C | 11 | 45216 | 45226 | *ycf3* |
| 26 | p1 | spacer | LSC | T | 10 | 45601 | 45610 | *ycf3-trnS(GGA)* |
| 27 | p1 | spacer | LSC | G | 10 | 47798 | 47807 | *rps4-trnL(CAA)* |
| 28 | p1 | spacer | LSC | A | 10 | 51902 | 51911 | *trnV(UAC)-trnM(CAU)* |
| 29 | p1 | spacer | LSC | T | 11 | 56710 | 56720 | *trnR(UCG)-accD* |
| 30 | p4 | spacer | LSC | TATT | 12 | 57765 | 57776 | *accD-psaI* |
| 31 | p2 | spacer | LSC | AT | 18 | 58231 | 58248 | *accD-psaI* |
| 32 | p2 | spacer | LSC | TA | 12 | 62570 | 62581 | *petA-psbJ* |
| 33 | p1 | spacer | LSC | A | 10 | 68052 | 68061 | *rpl20-rps12* |
| 34 | p1 | spacer | LSC | T | 11 | 68120 | 68130 | *rpl20-rps12* |
| 35 | p4 | spacer | LSC | GATA | 12 | 69048 | 69059 | *rps12-clpP* |
| 36 | p1 | intron | LSC | C | 11 | 69574 | 69584 | *chlP* |
| 37 | p1 | intron | LSC | T | 11 | 69615 | 69625 | *chlP* |
| 38 | p1 | intron | LSC | T | 11 | 70413 | 70423 | *chlP* |
| 39 | p2 | intron | LSC | CT | 12 | 70704 | 70715 | *chlP* |
| 40 | p2 | spacer | LSC | AT | 12 | 79990 | 80001 | *rps8-rpl14* |
| 41 | p1 | intron | LSC | T | 12 | 81564 | 81575 | *rpl16* |
| 42 | p1 | spacer | LSC | T | 10 | 85769 | 85778 | *rpl23-trnI(CAU)* |
| 43 | p2 | spacer | IRA | AT | 12 | 87653 | 87664 | *trnT(UGU)* |
| 44 | p4 | spacer | IRA | TCTT | 12 | 96474 | 96485 | *rrn16-rps12* |
| 45 | p1 | spacer | IRA | A | 12 | 97573 | 97584 | *rrn16-rps12* |
| 46 | p1 | spacer | SSC | A | 10 | 111684 | 111693 | *ndhF-rpl21* |
| 47 | p3 | spacer | SSC | AAT | 12 | 112846 | 112857 | *rpl32-trnP(GGG)* |
| 48 | p2 | spacer | SSC | TA | 12 | 112860 | 112871 | *rpl32-trnP(GGG)* |
| 49 | p3 | spacer | SSC | TAT | 12 | 112873 | 112884 | *rpl32-trnP(GGG)* |
| 50 | p1 | spacer | SSC | A | 10 | 113225 | 113234 | *trnP(GGG)-trnL(UAG)* |
| 51 | p1 | spacer | SSC | T | 11 | 113467 | 113477 | *trnL(UAG)-ccsA* |
| 52 | p1 | spacer | SSC | A | 10 | 116602 | 116611 | *ndhD-psaC* |
| 53 | p4 | spacer | SSC | AATT | 12 | 116610 | 116621 | *ndhD-psaC* |
| 54 | p1 | spacer | SSC | G | 11 | 117154 | 117164 | *psaC-ndhE* |
| 55 | p1 | spacer | SSC | T | 11 | 118927 | 118937 | *ndhI-ndhA* |
| 56 | p2 | spacer | SSC | AT | 16 | 118966 | 118981 | *ndhI-ndhA* |
| 57 | p1 | intron | SSC | T | 17 | 120503 | 120519 | *ndhA* |
| 58 | p1 | spacer | SSC | A | 10 | 122795 | 122804 | *rps15-ycf1* |
| 59 | p1 | spacer | IRB | T | 12 | 142438 | 142449 | *rps12-rrn16* |
| 60 | p4 | spacer | IRB | AAAG | 12 | 143536 | 143547 | *rps12-rrn16* |
| 61 | p2 | spacer | IRB | AT | 12 | 152358 | 152369 | *trnT(UGU)* |

Appendix Table S2 List of simple sequence repeats (SSRs) in the chloroplast genomes of *Gymnospaera podophylla*

| SSR nr. | SSR type | Locus | Region | SSR motif | size | start | end | Location |
| --- | --- | --- | --- | --- | --- | --- | --- | --- |
| 1 | p1 | spacer | LSC | G | 13 | 2113 | 2125 | *ndhB-matK* |
| 2 | p1 | intron | LSC | T | 10 | 5156 | 5165 | *rps16* |
| 3 | p1 | spacer | LSC | G | 16 | 7667 | 7682 | *chlB-trnQ(UUG)* |
| 4 | p2 | spacer | LSC | AT | 14 | 9108 | 9121 | *trnS(GUC)-psaM* |
| 5 | p1 | spacer | LSC | G | 11 | 9713 | 9723 | *ycf12-trnG(UCC)* |
| 6 | p4 | intron | LSC | AATT | 12 | 10328 | 10339 | *trnG(UCC)* |
| 7 | p1 | spacer | LSC | C | 14 | 14948 | 14961 | *atpH-atpI* |
| 8 | p1 | spacer | LSC | C | 11 | 15898 | 15908 | *atpI-rps2* |
| 9 | p1 | spacer | LSC | C | 11 | 27484 | 27494 | *rpoB-trnD(GUC)* |
| 10 | p4 | spacer | LSC | ATAG | 12 | 28409 | 28420 | *rpoB-trnD(GUC)* |
| 11 | p1 | spacer | LSC | T | 12 | 29103 | 29114 | *trnE(UUC)-psbM* |
| 12 | p4 | spacer | LSC | TGAT | 12 | 29197 | 29208 | *trnE(UUC)-psbM* |
| 13 | p1 | spacer | LSC | G | 13 | 30325 | 30337 | *ycf66* |
| 14 | p1 | spacer | LSC | A | 10 | 32400 | 32409 | *trnC(GCA)-trnG(GCC)* |
| 15 | p1 | spacer | LSC | C | 12 | 32787 | 32798 | *trnG(GCC)-psbZ* |
| 16 | p4 | spacer | LSC | TATC | 16 | 34223 | 34238 | *trnS(UGA)-psbC* |
| 17 | p4 | spacer | LSC | ATAG | 12 | 34242 | 34253 | *trnS(UGA)-psbC* |
| 18 | p1 | spacer | LSC | T | 10 | 37895 | 37904 | *psbD-trnT(GGU)* |
| 19 | p5 | intron | LSC | TTCTC | 15 | 39317 | 39331 | *rps14* |
| 20 | p3 | intron | LSC | AAT | 12 | 44793 | 44819 | *ycf3* |
| 21 | p1 | intron | LSC | T | 10 | 44810 | 44819 | *ycf3* |
| 22 | p1 | intron | LSC | C | 11 | 45899 | 45909 | *ycf3* |
| 23 | p1 | spacer | LSC | T | 10 | 46284 | 46293 | *ycf3-trnS(GGA)* |
| 24 | p1 | spacer | LSC | T | 10 | 46867 | 46876 | *trnS(GGA)-rps4* |
| 25 | p1 | spacer | LSC | G | 12 | 48604 | 48615 | *rps4-trnL(CAA)* |
| 26 | p1 | spacer | LSC | T | 10 | 57501 | 57510 | *trnR(UCG)-accD* |
| 27 | p4 | spacer | LSC | TATT | 12 | 58555 | 58566 | *accD-psaI* |
| 28 | p1 | spacer | LSC | A | 10 | 58615 | 58624 | *accD-psaI* |
| 29 | p2 | spacer | LSC | AT | 18 | 59022 | 59039 | *accD-psaI* |
| 30 | p2 | spacer | LSC | TA | 14 | 63376 | 63389 | *petA-psbJ* |
| 31 | p1 | spacer | LSC | T | 10 | 66579 | 66588 | *trnW(CCA)-trnP(UGG)* |
| 32 | p1 | spacer | LSC | C | 12 | 67291 | 67302 | *psaJ-rpl33* |
| 33 | p1 | spacer | LSC | A | 10 | 68892 | 68901 | *rpl20-rps12* |
| 34 | p1 | spacer | LSC | T | 11 | 68960 | 68970 | *rpl20-rps12* |
| 35 | p4 | spacer | LSC | GATA | 12 | 69890 | 69901 | *rps12-clpP* |
| 36 | p1 | intron | LSC | T | 10 | 70443 | 70452 | *clpP* |
| 37 | p1 | intron | LSC | T | 11 | 71239 | 71249 | *clpP* |
| 38 | p1 | intron | LSC | T | 11 | 75002 | 75012 | *petB* |
| 39 | p1 | spacer | LSC | T | 10 | 77981 | 77990 | *petD-rpoA* |
| 40 | p1 | spacer | LSC | A | 10 | 79921 | 79930 | *rpl36-infA* |
| 41 | p1 | spacer | LSC | T | 11 | 80751 | 80761 | *rps8-rpl14* |
| 42 | p1 | spacer | LSC | A | 10 | 80792 | 80801 | *rps8-rpl14* |
| 43 | p2 | spacer | LSC | AT | 16 | 80801 | 80816 | *rps8-rpl14* |
| 44 | p1 | intron | LSC | T | 13 | 82378 | 82390 | *rpl16* |
| 45 | p1 | intron | LSC | T | 10 | 82733 | 82742 | *rpl16* |
| 46 | p2 | spacer | IRB | AT | 12 | 87789 | 87800 | *trnT(UGU)* |
| 47 | p1 | spacer | IRB | A | 10 | 94889 | 94898 | *trnI(GAU)-rrn16* |
| 48 | p1 | spacer | IRB | A | 12 | 104005 | 104016 | *rrn16-rps12* |
| 49 | p1 | spacer | IRB | A | 10 | 107991 | 108000 | *trnH(GUG)-ycf2* |
| 50 | p1 | spacer | IRB | C | 10 | 115418 | 115427 | *trnN(GUU)-ndhF* |
| 51 | p1 | spacer | SSC | A | 10 | 117925 | 117934 | *ndhF-rpl21* |
| 52 | p2 | spacer | SSC | TA | 22 | 119288 | 119309 | *rpl32-trnP(GGG)* |
| 53 | p4 | spacer | SSC | AAAT | 12 | 119665 | 119676 | *trnP(GGG)-trnL(UAG)* |
| 54 | p1 | spacer | SSC | T | 13 | 119901 | 119913 | *trnL(UAG)-ccsA* |
| 55 | p1 | spacer | SSC | A | 10 | 123033 | 123042 | *ndhD-psaC* |
| 56 | p4 | spacer | SSC | AATT | 12 | 123041 | 123052 | *ndhD-psaC* |
| 57 | p1 | spacer | SSC | G | 15 | 123587 | 123601 | *psaC-ndhE* |
| 58 | p1 | spacer | SSC | T | 10 | 125374 | 125383 | *ndhI-ndhA* |
| 59 | p2 | spacer | SSC | AT | 14 | 125418 | 125431 | *ndhI-ndhA* |
| 60 | p2 | intron | SSC | TA | 16 | 126454 | 126469 | *ndhA* |
| 61 | p1 | intron | SSC | T | 17 | 126960 | 126976 | *ndhA* |
| 62 | p1 | spacer | SSC | C | 11 | 134606 | 134616 | *ycf1-chlN* |
| 63 | p1 | spacer | IRA | G | 10 | 137487 | 137496 | *chlL-trnN(GUU)* |
| 64 | p1 | spacer | IRA | T | 10 | 144914 | 144923 | *ycf2-trnH(GUG)* |
| 65 | p1 | spacer | IRA | T | 12 | 148898 | 148909 | *rps12-rrn16* |
| 66 | p1 | spacer | IRA | T | 10 | 158016 | 158025 | *rrn16-trnI(GAU)* |
| 67 | p2 | spacer | IRA | AT | 12 | 165114 | 165125 | *trnT(UGU)* |

Appendix Table S3 List of simple sequence repeats (SSRs) in the chloroplast genomes of *Gymnospaera gigantea*

| SSR nr. | SSR type | Locus | Region | SSR motif | size | star | end | Location |
| --- | --- | --- | --- | --- | --- | --- | --- | --- |
| 1 | p1 | spacer | LSC | G | 10 | 3677 | 3686 | *matK-rps16* |
| 2 | p1 | spacer | LSC | G | 11 | 7015 | 7025 | *chlB-trnQ(UUG)* |
| 3 | p2 | spacer | LSC | AT | 14 | 8451 | 8464 | *trnS(GUC)-psaM* |
| 4 | p4 | intron | LSC | AATT | 12 | 9622 | 9633 | *trnG(UCC)* |
| 5 | p1 | spacer | LSC | C | 10 | 14251 | 14260 | *atpH-atpI* |
| 6 | p1 | spacer | LSC | C | 10 | 15197 | 15206 | *atpI-rps2* |
| 7 | p1 | intron | LSC | C | 10 | 22460 | 22469 | *rpoC1* |
| 8 | p1 | spacer | LSC | T | 10 | 27192 | 27201 | *rpoB-trnD(GUC)* |
| 9 | p4 | spacer | LSC | ATAG | 12 | 27704 | 27715 | *rpoB-trnD(GUC)* |
| 10 | p4 | spacer | LSC | TGAT | 12 | 28490 | 28501 | *trnE(UUC)-psbM* |
| 11 | p1 | spacer | LSC | G | 17 | 29622 | 29638 | *ycf66* |
| 12 | p1 | spacer | LSC | G | 11 | 31628 | 31638 | *trnC(GCA)-trnG(GCC)* |
| 13 | p1 | spacer | LSC | C | 10 | 32088 | 32097 | *trnG(GCC)-psbZ* |
| 14 | p4 | spacer | LSC | TATC | 12 | 33522 | 33533 | *trnS(UGA)-psbC* |
| 15 | p4 | spacer | LSC | ATAG | 12 | 33537 | 33548 | *trnS(UGA)-psbC* |
| 16 | p1 | spacer | LSC | T | 10 | 36642 | 36651 | *psbD-trnT(GGU)* |
| 17 | p2 | spacer | LSC | AG | 24 | 36747 | 36770 | *psbD-trnT(GGU)* |
| 18 | p1 | intron | LSC | A | 10 | 44100 | 44109 | *ycf3* |
| 19 | p1 | intron | LSC | T | 12 | 44122 | 44133 | *ycf3* |
| 20 | p1 | spacer | LSC | T | 10 | 45598 | 45607 | *ycf3-trnS(GGA)* |
| 21 | p1 | spacer | LSC | G | 10 | 47441 | 47450 | *rps4-trnL(CAA)* |
| 22 | p1 | spacer | LSC | G | 10 | 47936 | 47945 | *rps4-trnL(CAA)* |
| 23 | p1 | spacer | LSC | A | 14 | 48913 | 48926 | *trnL(CAA)-trnF(GAA)* |
| 24 | p1 | spacer | LSC | T | 10 | 49833 | 49842 | *ndhJ-ndhK* |
| 25 | p1 | spacer | LSC | A | 11 | 52073 | 52083 | *trnV(UAC)-trnM(CAU)* |
| 26 | p1 | spacer | LSC | T | 11 | 54541 | 54551 | *atpB-rbcL* |
| 27 | p1 | spacer | LSC | T | 10 | 56891 | 56900 | *trnR(UCG)-accD* |
| 28 | p4 | spacer | LSC | TATT | 12 | 57947 | 57958 | *accD-psaI* |
| 29 | p2 | spacer | LSC | AT | 14 | 58413 | 58426 | *accD-psaI* |
| 30 | p2 | spacer | LSC | TA | 16 | 62759 | 62774 | *petA-psbJ* |
| 31 | p1 | spacer | LSC | C | 11 | 72815 | 72825 | *psaJ-rpl33* |
| 32 | p1 | spacer | LSC | A | 11 | 74414 | 74424 | *rpl20-rps12* |
| 33 | p1 | spacer | LSC | T | 10 | 74483 | 74492 | *rpl20-rps12* |
| 34 | p4 | spacer | LSC | GATA | 12 | 75418 | 75429 | *rps12-clpP* |
| 35 | p1 | intron | LSC | C | 11 | 75940 | 75950 | *clpP* |
| 36 | p1 | intron | LSC | T | 10 | 75981 | 75990 | *clpP* |
| 37 | p1 | intron | LSC | T | 11 | 81166 | 81176 | *petB* |
| 38 | p1 | spacer | LSC | A | 11 | 86310 | 86320 | *rps8-rpl14* |
| 39 | p2 | spacer | LSC | AT | 16 | 86320 | 86335 | *rps8-rpl14* |
| 40 | p1 | spacer | LSC | T | 11 | 86335 | 86345 | *rps8-rpl14* |
| 41 | p1 | intron | LSC | T | 12 | 88251 | 88262 | *rpl16* |
| 42 | p1 | intron | LSC | C | 10 | 91441 | 91450 | *rpl2* |
| 43 | p2 | spacer | IRB | AT | 12 | 93990 | 94001 | *trnT(UGU)* |
| 44 | p1 | spacer | IRB | A | 12 | 101110 | 101121 | *trnI(GAU)-rrn16* |
| 45 | p4 | spacer | IRB | TCTT | 12 | 103414 | 103425 | *rrn16-rps12* |
| 46 | p1 | spacer | IRB | A | 12 | 104529 | 104540 | *rrn16-rps12* |
| 47 | p3 | spacer | SSC | AAT | 12 | 119784 | 119795 | *rpl32-trnP(GGG)* |
| 48 | p2 | spacer | SSC | TA | 18 | 119798 | 119815 | *rpl32-trnP(GGG)* |
| 49 | p3 | spacer | SSC | TAT | 12 | 119817 | 119828 | *rpl32-trnP(GGG)* |
| 50 | p4 | spacer | SSC | GAAA | 12 | 120167 | 120178 | *trnP(GGG)-trnL(UAG)* |
| 51 | p1 | spacer | SSC | T | 13 | 120409 | 120421 | *trnL(UAG)-ccsA* |
| 52 | p1 | spacer | SSC | A | 11 | 121680 | 121690 | *ccsA-ndhD* |
| 53 | p1 | spacer | SSC | A | 13 | 123545 | 123557 | *ndhD-psaC* |
| 54 | p1 | spacer | SSC | T | 10 | 123566 | 123575 | *ndhD-psaC* |
| 55 | p1 | spacer | SSC | G | 31 | 124104 | 124134 | *psaC-ndhE* |
| 56 | p1 | spacer | SSC | A | 24 | 125949 | 125961 | *ndhI-ndhA* |
| 57 | p2 | spacer | SSC | AT | 12 | 125961 | 125972 | *ndhI-ndhA* |
| 58 | p2 | intron | SSC | TA | 14 | 126999 | 127012 | *ndhA* |
| 59 | p1 | spacer | SSC | C | 31 | 135145 | 135175 | *ycf1-chlN* |
| 60 | p4 | spacer | SSC | AAAT | 16 | 135394 | 135409 | *ycf1-chlN* |
| 61 | p1 | spacer | IRA | T | 12 | 149455 | 149466 | *rps12-rrn16* |
| 62 | p4 | spacer | IRA | AAAG | 12 | 150569 | 150580 | *rps12-rrn16* |
| 63 | p1 | spacer | IRA | T | 12 | 152874 | 152885 | *rrn16-trnI(GAU)* |
| 64 | p2 | spacer | IRA | AT | 12 | 159994 | 160005 | *trnT(UGU)* |

Appendix Table S4 List of simple sequence repeats (SSRs) in the chloroplast genomes of *Gymnospaera metteniana*

| SSR nr. | SSR type | Locus | Region | SSR motif | size | star | end | Location |
| --- | --- | --- | --- | --- | --- | --- | --- | --- |
| 1 | p1 | spacer | LSC | G | 12 | 7094 | 7105 | *chlB-trnQ(UUG)* |
| 2 | p1 | spacer | LSC | T | 10 | 8416 | 8425 | *trnS(GUC)-psaM* |
| 3 | p2 | spacer | LSC | AT | 14 | 8532 | 8545 | *trnS(GUC)-psaM* |
| 4 | p4 | intron | LSC | AATT | 12 | 9703 | 9714 | *trnG(UCC)* |
| 5 | p1 | intron | LSC | C | 10 | 22538 | 22547 | *trnG(UCC)* |
| 6 | p1 | spacer | LSC | T | 10 | 27270 | 27279 | *rpoB-trnD(GUC)* |
| 7 | p4 | spacer | LSC | ATAG | 12 | 27782 | 27793 | *rpoB-trnD(GUC)* |
| 8 | p4 | spacer | LSC | TGAT | 12 | 28568 | 28579 | *trnE(UUC)-psbM* |
| 9 | p1 | spacer | LSC | G | 14 | 29700 | 29713 | *ycf66* |
| 10 | p1 | spacer | LSC | G | 11 | 31714 | 31724 | *trnC(GCA)-trnG(GCC)* |
| 11 | p1 | spacer | LSC | C | 12 | 32174 | 32185 | *trnG(GCC)-psbZ* |
| 12 | p4 | spacer | LSC | TATC | 12 | 33610 | 33621 | *trnS(UGA)-psbC* |
| 13 | p4 | spacer | LSC | ATAG | 12 | 33625 | 33636 | *trnS(UGA)-psbC* |
| 14 | p1 | spacer | LSC | T | 10 | 36730 | 36739 | *psbD-trnT(GGU)* |
| 15 | p2 | spacer | LSC | AG | 16 | 36835 | 36850 | *psbD-trnT(GGU)* |
| 16 | p1 | intron | LSC | T | 13 | 44201 | 44213 | *ycf3* |
| 17 | p1 | spacer | LSC | T | 10 | 45678 | 45687 | *ycf3-trnS(GGA)* |
| 18 | p1 | spacer | LSC | G | 10 | 47521 | 47530 | *rps4-trnL(CAA)* |
| 19 | p1 | spacer | LSC | G | 10 | 48027 | 48036 | *rps4-trnL(CAA)* |
| 20 | p1 | spacer | LSC | A | 13 | 48988 | 49000 | *trnL(CAA)-trnF(GAA)* |
| 21 | p1 | spacer | LSC | T | 10 | 49907 | 49916 | *ndhJ-ndhK* |
| 22 | p1 | spacer | LSC | A | 10 | 52147 | 52156 | *trnV(UAC)-trnM(CAU)* |
| 23 | p1 | spacer | LSC | T | 11 | 54614 | 54624 | *atpB-rbcL* |
| 24 | p1 | spacer | LSC | T | 10 | 56964 | 56973 | *trnR(UCG)-accD* |
| 25 | p4 | spacer | LSC | TATT | 12 | 58020 | 58031 | *accD-psaI* |
| 26 | p2 | spacer | LSC | AT | 14 | 58486 | 58499 | *accD-psaI* |
| 27 | p2 | spacer | LSC | TA | 16 | 62833 | 62848 | *petA-psbJ* |
| 28 | p5 | spacer | LSC | CTTTC | 15 | 71752 | 71766 | *petG-trnW(CCA)* |
| 29 | p1 | spacer | LSC | C | 12 | 72879 | 72890 | *psaJ-rpl33* |
| 30 | p1 | spacer | LSC | A | 10 | 74479 | 74488 | *rpl20-rps12* |
| 31 | p1 | spacer | LSC | T | 10 | 74547 | 74556 | *rpl20-rps12* |
| 32 | p4 | spacer | LSC | GATA | 12 | 75482 | 75493 | *rps12-clpP* |
| 33 | p1 | intron | LSC | C | 10 | 76004 | 76013 | *clpP* |
| 34 | p1 | intron | LSC | T | 14 | 76044 | 76057 | *clpP* |
| 35 | p1 | intron | LSC | T | 10 | 81233 | 81242 | *petB* |
| 36 | p2 | spacer | LSC | AT | 18 | 86384 | 86401 | *rps8-rpl14* |
| 37 | p1 | intron | LSC | T | 11 | 88315 | 88325 | *rpl16* |
| 38 | p1 | intron | LSC | C | 10 | 91506 | 91515 | *rpl2* |
| 39 | p2 | spacer | IRB | AT | 12 | 94055 | 94066 | *trnT(UGU)* |
| 40 | p1 | spacer | IRB | A | 12 | 101175 | 101186 | *trnI(GAU)-rrn16* |
| 41 | p4 | spacer | IRB | TCTT | 12 | 103479 | 103490 | *rrn16-rps12* |
| 42 | p1 | spacer | IRB | A | 14 | 104578 | 104591 | *rrn16-rps12* |
| 43 | p1 | spacer | IRB | T | 10 | 106780 | 106789 | *rps7-psbA* |
| 44 | p3 | spacer | SSC | AAT | 12 | 119836 | 119847 | *rpl32-trnP(GGG)* |
| 45 | p2 | spacer | SSC | TA | 18 | 119850 | 119867 | *rpl32-trnP(GGG)* |
| 46 | p3 | spacer | SSC | TAT | 12 | 119869 | 119880 | *rpl32-trnP(GGG)* |
| 47 | p4 | spacer | SSC | GAAA | 12 | 120219 | 120230 | *trnP(GGG)-trnL(UAG)* |
| 48 | p1 | spacer | SSC | T | 12 | 120461 | 120472 | *trnL(UAG)-ccsA* |
| 49 | p1 | spacer | SSC | A | 11 | 121731 | 121741 | *ccsA-ndhD* |
| 50 | p1 | spacer | SSC | A | 11 | 123596 | 123606 | *ndhD-psaC* |
| 51 | p4 | spacer | SSC | AATT | 12 | 123605 | 123616 | *ndhD-psaC* |
| 52 | p1 | spacer | SSC | T | 10 | 123615 | 123624 | *ndhD-psaC* |
| 53 | p1 | spacer | SSC | G | 15 | 124153 | 124167 | *psaC-ndhE* |
| 54 | p1 | spacer | SSC | A | 24 | 125982 | 125992 | *ndhI-ndhA* |
| 55 | p2 | spacer | SSC | AT | 14 | 125992 | 126005 | *ndhI-ndhA* |
| 56 | p1 | spacer | SSC | A | 13 | 126041 | 126053 | *ndhI-ndhA* |
| 57 | p2 | intron | SSC | TA | 14 | 127030 | 127043 | *ndhA* |
| 58 | p1 | spacer | SSC | A | 11 | 129846 | 129856 | *rps15-ycf1* |
| 59 | p1 | spacer | SSC | C | 14 | 135178 | 135191 | *ycf1-chlN* |
| 60 | p4 | spacer | SSC | AAAT | 16 | 135410 | 135425 | *ycf1-chlN* |
| 61 | p1 | spacer | IRA | A | 10 | 147274 | 147283 | *psbA-rps7* |
| 62 | p1 | spacer | IRA | T | 14 | 149472 | 149485 | *rps12-rrn16* |
| 63 | p4 | spacer | IRA | AAAG | 12 | 150572 | 150583 | *rps12-rrn16* |
| 64 | p1 | spacer | IRA | T | 12 | 152877 | 152888 | *rrn16-trnI(GAU)* |
| 65 | p2 | spacer | IRA | AT | 12 | 159997 | 160008 | *trnT(UGU)* |

Appendix Table S5 List of simple sequence repeats (SSRs) in the chloroplast genomes of *Alsophila costularis*

| SSR nr. | SSR type | Locus | Region | SSR motif | size | star | end | Location |
| --- | --- | --- | --- | --- | --- | --- | --- | --- |
| 1 | p1 | intron | LSC | T | 11 | 367 | 374 | *ndhB* |
| 2 | p1 | spacer | LSC | A | 12 | 3443 | 3454 | *matK-rps16* |
| 3 | p1 | spacer | LSC | T | 10 | 3538 | 3547 | *matK-rps16* |
| 4 | p1 | intron | LSC | T | 11 | 4288 | 4298 | *rps16* |
| 5 | p1 | spacer | LSC | G | 15 | 6798 | 6812 | *chlB-trnQ(UUG)* |
| 6 | p1 | spacer | LSC | A | 11 | 7517 | 7527 | *psbK-psbI* |
| 7 | p2 | spacer | LSC | TA | 12 | 7529 | 7540 | *psbK-psbI* |
| 8 | p1 | spacer | LSC | C | 13 | 8126 | 8138 | *trnS(GCU)-psaM* |
| 9 | p2 | spacer | LSC | AT | 16 | 8229 | 8244 | *trnS(GCU)-psaM* |
| 10 | p1 | spacer | LSC | T | 10 | 8244 | 8253 | *trnS(GCU)-psaM* |
| 11 | p1 | spacer | LSC | G | 11 | 8797 | 8807 | *ycf12-trnG(UCC)* |
| 12 | p1 | intron | LSC | C | 14 | 9681 | 9694 | *trnG(UCC)* |
| 13 | p1 | spacer | LSC | C | 19 | 14047 | 14065 | *atpH-atpI* |
| 14 | p1 | spacer | LSC | A | 11 | 14078 | 14088 | *atpH-atpI* |
| 15 | p1 | spacer | LSC | T | 13 | 15006 | 15018 | *atpI-rps2* |
| 16 | p1 | intron | LSC | T | 13 | 22288 | 22300 | *rpoC1* |
| 17 | p3 | intron | LSC | AAG | 12 | 22479 | 22490 | *rpoC1* |
| 18 | p1 | spacer | LSC | A | 11 | 23235 | 23245 | *rpoC1-rpoB* |
| 19 | p1 | spacer | LSC | T | 10 | 27599 | 27608 | *rpoB-trnD(GUC)* |
| 20 | p1 | spacer | LSC | A | 12 | 28205 | 28216 | *trnE(UUC)-psbM* |
| 21 | p1 | spacer | LSC | A | 12 | 29369 | 29380 | *ycf66* |
| 22 | p2 | spacer | LSC | AT | 18 | 30361 | 30378 | *petN-trnC(GCA)* |
| 23 | p1 | spacer | LSC | G | 12 | 31701 | 31712 | *trnC(GCA)-trnG(GCC)* |
| 24 | p1 | spacer | LSC | A | 13 | 31782 | 31794 | *trnC(GCA)-trnG(GCC)* |
| 25 | p1 | spacer | LSC | A | 12 | 32996 | 33007 | *trnS(UGA)-psbC* |
| 26 | p1 | spacer | LSC | T | 13 | 33067 | 33079 | *trnS(UGA)-psbC* |
| 27 | p4 | spacer | LSC | ATCT | 16 | 33600 | 33615 | *trnS(UGA)-psbC* |
| 28 | p4 | spacer | LSC | ATAG | 12 | 33618 | 33629 | *trnS(UGA)-psbC* |
| 29 | p1 | spacer | LSC | T | 11 | 36726 | 36736 | *psbD-trnT(GGU)* |
| 30 | p1 | spacer | LSC | A | 10 | 36854 | 36863 | *psbD-trnT(GGU)* |
| 31 | p1 | spacer | LSC | T | 19 | 37272 | 37290 | *psbD-trnT(GGU)* |
| 32 | p1 | spacer | LSC | A | 12 | 37670 | 37681 | *psbD-trnT(GGU)* |
| 33 | p1 | intron | LSC | T | 11 | 44231 | 44241 | *ycf3* |
| 34 | p1 | intron | LSC | C | 11 | 45328 | 45338 | *ycf3* |
| 35 | p1 | spacer | LSC | T | 15 | 45725 | 45739 | *ycf3-trnS(GGA)* |
| 36 | p1 | spacer | LSC | A | 10 | 45781 | 45790 | *ycf3-trnS(GGA)* |
| 37 | p1 | spacer | LSC | T | 12 | 45851 | 45862 | *ycf3-trnS(GGA)* |
| 38 | p1 | spacer | LSC | T | 11 | 46321 | 46331 | *trnS(GGA)-rps4* |
| 39 | p1 | spacer | LSC | G | 10 | 48024 | 48033 | *rps4-trnL(CAA)* |
| 40 | p1 | intron | LSC | A | 14 | 48342 | 48355 | *trnL(CAA)* |
| 41 | p1 | spacer | LSC | T | 14 | 48944 | 48957 | *trnL(CAA)-trnF(GAA)* |
| 42 | p1 | spacer | LSC | T | 10 | 51286 | 51295 | *ndhC-trnV(UAC)* |
| 43 | p1 | spacer | LSC | A | 11 | 51302 | 51312 | *ndhC-trnV(UAC)* |
| 44 | p1 | spacer | LSC | A | 10 | 52147 | 52156 | *trnV(UAC)-trnM(CAU)* |
| 45 | p2 | spacer | LSC | AT | 14 | 52242 | 52255 | *trnV(UAC)-trnM(CAU)* |
| 46 | p1 | spacer | LSC | T | 13 | 54712 | 54724 | *atpB-rbcL* |
| 47 | p1 | spacer | LSC | T | 12 | 56557 | 56568 | *rbcL-trnR(UCG)* |
| 48 | p1 | spacer | LSC | T | 19 | 56978 | 56996 | *trnR(UCG)-accD* |
| 49 | p4 | spacer | LSC | TATT | 12 | 58025 | 58036 | *accD-psaI* |
| 50 | p1 | spacer | LSC | A | 10 | 58085 | 58094 | *accD-psaI* |
| 51 | p1 | spacer | LSC | T | 10 | 58508 | 58517 | *accD-psaI* |
| 52 | p1 | spacer | LSC | C | 11 | 62532 | 62542 | *petA-psbJ* |
| 53 | p1 | spacer | LSC | T | 13 | 63161 | 63173 | *petA-psbJ* |
| 54 | p1 | spacer | LSC | T | 11 | 64725 | 64735 | *psbE-petL* |
| 55 | p1 | spacer | LSC | T | 10 | 65541 | 65550 | *petG-trnW(CCA)* |
| 56 | p1 | spacer | LSC | T | 10 | 65940 | 65949 | *trnW(CCA)-trnP(UGG)* |
| 57 | p1 | spacer | LSC | T | 18 | 67335 | 67352 | *rps18-rpl20* |
| 58 | p1 | spacer | LSC | T | 12 | 68310 | 68321 | *rpl20-rps12* |
| 59 | p1 | spacer | LSC | T | 11 | 68513 | 68523 | *rpl20-rps12* |
| 60 | p1 | spacer | LSC | T | 11 | 68770 | 68780 | *rpl20-rps12* |
| 61 | p4 | spacer | LSC | TATC | 12 | 69226 | 69237 | *rps12-clpP* |
| 62 | p4 | spacer | LSC | GATA | 12 | 69244 | 69255 | *rps12-clpP* |
| 63 | p1 | intron | LSC | C | 42 | 69761 | 69802 | *clpP* |
| 64 | p1 | intron | LSC | C | 10 | 70520 | 70529 | *clpP* |
| 65 | p2 | intron | LSC | AT | 24 | 70655 | 70678 | *clpP* |
| 66 | p1 | spacer | LSC | A | 11 | 71659 | 71669 | *clpP-psbB* |
| 67 | p1 | intron | LSC | T | 16 | 74446 | 74461 | *petB* |
| 68 | p1 | intron | LSC | T | 10 | 76585 | 76594 | *petD* |
| 69 | p1 | spacer | LSC | T | 13 | 80192 | 80204 | *rps8-rpl14* |
| 70 | p2 | spacer | LSC | AT | 18 | 80224 | 80241 | *rps8-rpl14* |
| 71 | p1 | spacer | LSC | A | 14 | 80308 | 80321 | *rps8-rpl14* |
| 72 | p2 | spacer | LSC | TA | 12 | 80322 | 80333 | *rps8-rpl14* |
| 73 | p1 | spacer | LSC | T | 12 | 80342 | 80353 | *rps8-rpl14* |
| 74 | p1 | intron | LSC | T | 12 | 81928 | 81939 | *rpl16* |
| 75 | p4 | intron | LSC | CCTC | 12 | 82094 | 82105 | *rpl16* |
| 76 | p1 | intron | LSC | T | 13 | 82267 | 82279 | *rpl16* |
| 77 | p1 | spacer | LSC | T | 10 | 82493 | 82502 | *rpl16-rps3* |
| 78 | p1 | intron | LSC | A | 14 | 84696 | 84709 | *rpl2* |
| 79 | p1 | spacer | LSC | A | 12 | 85684 | 85695 | *rpl2-rpl23* |
| 80 | p1 | spacer | LSC | T | 13 | 85997 | 86009 | *rpl23-trnI(CAU)* |
| 81 | p1 | spacer | LSC | T | 11 | 86139 | 86149 | *rpl23-trnI(CAU)* |
| 82 | p2 | spacer | IRB | AT | 12 | 88021 | 88032 | *trnT(UGU)* |
| 83 | p2 | spacer | IRB | AT | 12 | 88476 | 88487 | *trnT(UGU)-trnR(ACG)* |
| 84 | p1 | spacer | IRB | T | 13 | 89923 | 89935 | *trnR(ACG)-rrn5* |
| 85 | p1 | spacer | IRB | C | 13 | 97451 | 97463 | *rrn16-rps12* |
| 86 | p4 | spacer | IRB | TCTT | 12 | 97984 | 97995 | *rrn16-rps12* |
| 87 | p1 | spacer | IRB | T | 11 | 99097 | 99107 | *rrn16-rps12* |
| 88 | p1 | spacer | IRB | A | 12 | 103055 | 103066 | *trnH(GUG)-ycf2* |
| 89 | p4 | CDS | IRB | AGAA | 12 | 108231 | 108242 | *ycf2* |
| 90 | p1 | spacer | IRB | A | 17 | 109641 | 109657 | *ycf2-trnN(GUU)* |
| 91 | p1 | spacer | IRB | G | 17 | 110427 | 110443 | *trnN(GUU)-ndhF* |
| 92 | p1 | spacer | SSC | T | 10 | 113600 | 113609 | *rpl21-rpl32* |
| 93 | p1 | spacer | SSC | A | 13 | 113713 | 113725 | *rpl21-rpl32* |
| 94 | p2 | spacer | SSC | AT | 16 | 113725 | 113740 | *rpl21-rpl32* |
| 95 | p1 | spacer | SSC | A | 12 | 114078 | 114089 | *rpl32-trnP(GGG)* |
| 96 | p1 | spacer | SSC | G | 10 | 114333 | 114342 | *rpl32-trnP(GGG)* |
| 97 | p1 | spacer | SSC | T | 14 | 114400 | 114413 | *rpl32-trnP(GGG)* |
| 98 | p1 | spacer | SSC | A | 13 | 114740 | 114752 | *trnP(GGG)-trnL(UAG)* |
| 99 | p1 | spacer | SSC | A | 11 | 114900 | 114910 | *trnL(UAG)-ccsA* |
| 100 | p1 | spacer | SSC | T | 12 | 114979 | 114990 | *trnL(UAG)-ccsA* |
| 101 | p1 | spacer | SSC | A | 11 | 118132 | 118142 | *ndhD-psaC* |
| 102 | p1 | spacer | SSC | T | 12 | 118147 | 118158 | *ndhD-psaC* |
| 103 | p1 | spacer | SSC | G | 13 | 118694 | 118706 | *psaC-ndhE* |
| 104 | p2 | spacer | SSC | TA | 14 | 120509 | 120522 | *ndhI-ndhA* |
| 105 | p1 | spacer | SSC | A | 13 | 120526 | 120538 | *ndhI-ndhA* |
| 106 | p2 | spacer | SSC | AT | 20 | 120538 | 120557 | *ndhI-ndhA* |
| 107 | p1 | spacer | SSC | A | 10 | 120602 | 120611 | *ndhI-ndhA* |
| 108 | p1 | intron | SSC | T | 10 | 121522 | 121531 | *ndhA* |
| 109 | p1 | spacer | SSC | A | 14 | 124314 | 124327 | *rps15-ycf1* |
| 110 | p4 | CDS | SSC | ACCA | 12 | 129098 | 129109 | *ycf1* |
| 111 | p1 | spacer | SSC | C | 13 | 129639 | 129651 | *ycf1-chlN* |
| 112 | p1 | spacer | IRA | C | 17 | 132571 | 132587 | *chlL-trnN(GUU)* |
| 113 | p1 | spacer | IRA | T | 17 | 133357 | 133373 | *trnN(GUU)-ycf2* |
| 114 | p4 | CDS | IRA | TTTC | 12 | 134771 | 134782 | *ycf2* |
| 115 | p1 | spacer | IRA | T | 12 | 139948 | 139959 | *ycf2-trnH(GUG)* |
| 116 | p1 | spacer | IRA | A | 11 | 143907 | 143917 | *rps12-rrn16* |
| 117 | p4 | spacer | IRA | AAAG | 12 | 145018 | 145029 | *rps12-rrn16* |
| 118 | p1 | spacer | IRA | G | 13 | 145551 | 145563 | *rps12-rrn16* |
| 119 | p1 | spacer | IRA | A | 13 | 153079 | 153091 | *rrn5-trnR(ACG)* |
| 120 | p2 | spacer | IRA | AT | 12 | 154527 | 154538 | *trnR(ACG)-trnT(UGU)* |
| 121 | p2 | spacer | IRA | AT | 12 | 154982 | 154993 | *trnT(UGU)* |

Appendix Table S6 List of simple sequence repeats (SSRs) in the chloroplast genomes of *Alsophila spinulosa*

| SSR nr. | SSR type | Locus | Region | SSR motif | size | star | end | Location |
| --- | --- | --- | --- | --- | --- | --- | --- | --- |
| 1 | p1 | intron | LSC | T | 11 | 364 | 374 | *ndhB* |
| 2 | p1 | spacer | LSC | A | 13 | 3443 | 3455 | *matK-rps16* |
| 3 | p1 | spacer | LSC | T | 10 | 3539 | 3548 | *matK-rps16* |
| 4 | p1 | intron | LSC | T | 11 | 4289 | 4299 | *rps16* |
| 5 | p1 | spacer | LSC | G | 14 | 6799 | 6812 | *chlB-trnQ(UUG)* |
| 6 | p1 | spacer | LSC | A | 24 | 7517 | 7527 | *psbK-psbI* |
| 7 | p2 | spacer | LSC | TA | 12 | 7529 | 7540 | *psbK-psbI* |
| 8 | p1 | spacer | LSC | C | 12 | 8126 | 8137 | *trnS(GCU)-psaM* |
| 9 | p2 | spacer | LSC | AT | 16 | 8228 | 8243 | *trnS(GCU)-psaM* |
| 10 | p1 | spacer | LSC | T | 10 | 8243 | 8252 | *trnS(GCU)-psaM* |
| 11 | p1 | spacer | LSC | G | 11 | 8796 | 8806 | *ycf12-trnG(UCC)* |
| 12 | p1 | intron | LSC | C | 13 | 9680 | 9692 | *trnG(UCC)* |
| 13 | p1 | spacer | LSC | C | 10 | 14045 | 14054 | *atpH-atpI* |
| 14 | p1 | spacer | LSC | A | 11 | 14072 | 14082 | *atpH-atpI* |
| 15 | p1 | spacer | LSC | T | 12 | 15000 | 15011 | *atpI-rps2* |
| 16 | p1 | intron | LSC | T | 13 | 22281 | 22293 | *rpoC1* |
| 17 | p3 | intron | LSC | AAG | 12 | 22472 | 22483 | *rpoC1* |
| 18 | p1 | spacer | LSC | A | 11 | 23228 | 23238 | *rpoC1-rpoB* |
| 19 | p1 | spacer | LSC | T | 10 | 27592 | 27601 | *rpoB-trnD(GUC)* |
| 20 | p1 | spacer | LSC | A | 12 | 28198 | 28209 | *trnE(UUC)-psbM* |
| 21 | p1 | spacer | LSC | A | 12 | 29362 | 29373 | *ycf66* |
| 22 | p2 | spacer | LSC | AT | 18 | 30354 | 30371 | *petN-trnC(GCA)* |
| 23 | p1 | spacer | LSC | G | 12 | 31694 | 31705 | *trnC(GCA)-trnG(GCC)* |
| 24 | p1 | spacer | LSC | A | 13 | 31775 | 31787 | *trnC(GCA)-trnG(GCC)* |
| 25 | p1 | spacer | LSC | A | 12 | 32989 | 33000 | *trnS(UGA)-psbC* |
| 26 | p1 | spacer | LSC | T | 13 | 33060 | 33072 | *trnS(UGA)-psbC* |
| 27 | p4 | spacer | LSC | ATCT | 16 | 33593 | 33608 | *trnS(UGA)-psbC* |
| 28 | p4 | spacer | LSC | ATAG | 12 | 33611 | 33622 | *trnS(UGA)-psbC* |
| 29 | p1 | spacer | LSC | T | 11 | 36719 | 36729 | *psbD-trnT(GGU)* |
| 30 | p1 | spacer | LSC | A | 10 | 36847 | 36856 | *psbD-trnT(GGU)* |
| 31 | p1 | spacer | LSC | T | 19 | 37265 | 37283 | *psbD-trnT(GGU)* |
| 32 | p1 | spacer | LSC | A | 11 | 37663 | 37673 | *psbD-trnT(GGU)* |
| 33 | p1 | intron | LSC | T | 12 | 44223 | 44234 | *ycf3* |
| 34 | p1 | intron | LSC | C | 11 | 45321 | 45331 | *ycf3* |
| 35 | p1 | spacer | LSC | T | 15 | 45718 | 45732 | *ycf3-trnS(GGA)* |
| 36 | p1 | spacer | LSC | A | 10 | 45774 | 45783 | *ycf3-trnS(GGA)* |
| 37 | p1 | spacer | LSC | T | 12 | 45844 | 45855 | *ycf3-trnS(GGA)* |
| 38 | p1 | spacer | LSC | T | 11 | 46314 | 46324 | *trnS(GGA)-rps4* |
| 39 | p1 | spacer | LSC | G | 10 | 48017 | 48026 | *rps4-trnL(CAA)* |
| 40 | p1 | intron | LSC | A | 14 | 48335 | 48348 | *trnL(CAA)* |
| 41 | p1 | spacer | LSC | T | 13 | 48937 | 48949 | *trnL(CAA)-trnF(GAA)* |
| 42 | p1 | spacer | LSC | T | 10 | 51278 | 51287 | *ndhC-trnV(UAC)* |
| 43 | p1 | spacer | LSC | A | 11 | 51294 | 51304 | *ndhC-trnV(UAC)* |
| 44 | p1 | spacer | LSC | A | 10 | 52139 | 52148 | *trnV(UAC)-trnM(CAU)* |
| 45 | p2 | spacer | LSC | AT | 14 | 52234 | 52247 | *trnV(UAC)-trnM(CAU)* |
| 46 | p1 | spacer | LSC | T | 13 | 54704 | 54716 | *atpB-rbcL* |
| 47 | p1 | spacer | LSC | T | 12 | 56549 | 56560 | *rbcL-trnR(UCG)* |
| 48 | p1 | spacer | LSC | T | 19 | 56970 | 56988 | *trnR(UCG)-accD* |
| 49 | p4 | spacer | LSC | TATT | 12 | 58017 | 58028 | *accD-psaI* |
| 50 | p1 | spacer | LSC | A | 10 | 58077 | 58086 | *accD-psaI* |
| 51 | p1 | spacer | LSC | T | 11 | 58500 | 58510 | *accD-psaI* |
| 52 | p1 | spacer | LSC | C | 11 | 62525 | 62535 | *petA-psbJ* |
| 53 | p1 | spacer | LSC | T | 13 | 63154 | 63166 | *petA-psbJ* |
| 54 | p1 | spacer | LSC | T | 11 | 64718 | 64728 | *psbE-petL* |
| 55 | p1 | spacer | LSC | T | 10 | 65534 | 65543 | *petG-trnW(CCA)* |
| 56 | p1 | spacer | LSC | T | 10 | 65933 | 65942 | *trnW(CCA)-trnP(UGG)* |
| 57 | p1 | spacer | LSC | C | 10 | 66640 | 66649 | *psaJ-rpl33* |
| 58 | p1 | spacer | LSC | T | 17 | 67329 | 67345 | *rps18-rpl20* |
| 59 | p1 | spacer | LSC | T | 12 | 68303 | 68314 | *rpl20-rps12* |
| 60 | p1 | spacer | LSC | T | 11 | 68506 | 68516 | *rpl20-rps12* |
| 61 | p1 | spacer | LSC | T | 11 | 68763 | 68773 | *rpl20-rps12* |
| 62 | p4 | spacer | LSC | TATC | 12 | 69219 | 69230 | *rps12-clpP* |
| 63 | p4 | spacer | LSC | GATA | 12 | 69237 | 69248 | *rps12-clpP* |
| 64 | p1 | intron | LSC | C | 17 | 69754 | 69770 | *clpP* |
| 65 | p1 | intron | LSC | C | 11 | 70488 | 70498 | *clpP* |
| 66 | p2 | intron | LSC | AT | 24 | 70624 | 70647 | *clpP* |
| 67 | p1 | spacer | LSC | A | 11 | 71628 | 71638 | *clpP-psbB* |
| 68 | p1 | intron | LSC | T | 16 | 74415 | 74430 | *petB* |
| 69 | p1 | intron | LSC | T | 10 | 76554 | 76563 | *petD* |
| 70 | p1 | spacer | LSC | T | 13 | 80161 | 80173 | *rps8-rpl14* |
| 71 | p2 | spacer | LSC | AT | 18 | 80193 | 80210 | *rps8-rpl14* |
| 72 | p1 | spacer | LSC | A | 14 | 80277 | 80290 | *rps8-rpl14* |
| 73 | p2 | spacer | LSC | TA | 12 | 80291 | 80302 | *rps8-rpl14* |
| 74 | p1 | spacer | LSC | T | 12 | 80311 | 80322 | *rps8-rpl14* |
| 75 | p1 | intron | LSC | T | 12 | 81897 | 81908 | *rpl16* |
| 76 | p4 | intron | LSC | CCTC | 12 | 82063 | 82074 | *rpl16* |
| 77 | p1 | intron | LSC | T | 13 | 82236 | 82248 | *rpl16* |
| 78 | p1 | spacer | LSC | T | 10 | 82462 | 82471 | *rpl16-rps3* |
| 79 | p1 | intron | LSC | A | 15 | 84665 | 84679 | *rpl2* |
| 80 | p1 | spacer | LSC | A | 12 | 85654 | 85665 | *rpl2-rpl23* |
| 81 | p1 | spacer | LSC | T | 13 | 85967 | 85979 | *rpl23-trnI(CAU)* |
| 82 | p1 | spacer | LSC | T | 11 | 86109 | 86119 | *rpl23-trnI(CAU)* |
| 83 | p2 | spacer | IRB | AT | 12 | 87991 | 88002 | *trnT(UGU)* |
| 84 | p2 | spacer | IRB | AT | 12 | 88446 | 88457 | *trnT(UGU)-trnR(ACG)* |
| 85 | p1 | spacer | IRB | T | 13 | 89893 | 89905 | *trnR(ACG)-rrn5* |
| 86 | p1 | spacer | IRB | C | 11 | 97421 | 97431 | *rrn16-rps12* |
| 87 | p4 | spacer | IRB | TCTT | 12 | 97952 | 97963 | *rrn16-rps12* |
| 88 | p1 | spacer | IRB | T | 11 | 99065 | 99075 | *rrn16-rps12* |
| 89 | p1 | spacer | IRB | A | 12 | 103023 | 103034 | *trnH(GUG)-ycf2* |
| 90 | p4 | CDS | IRB | AGAA | 12 | 108199 | 108210 | *ycf2* |
| 91 | p1 | spacer | IRB | A | 23 | 109609 | 109631 | *ycf2-trnN(GUU)* |
| 92 | p1 | spacer | IRB | G | 15 | 110408 | 110422 | *trnN(GUU)-ndhF* |
| 93 | p1 | spacer | SSC | T | 10 | 113579 | 113588 | *rpl21-rpl32* |
| 94 | p1 | spacer | SSC | A | 13 | 113692 | 113704 | *rpl21-rpl32* |
| 95 | p2 | spacer | SSC | AT | 16 | 113704 | 113719 | *rpl21-rpl32* |
| 96 | p1 | spacer | SSC | A | 11 | 114057 | 114067 | *rpl32-trnP(GGG)* |
| 97 | p1 | spacer | SSC | G | 10 | 114311 | 114320 | *rpl32-trnP(GGG)* |
| 98 | p1 | spacer | SSC | T | 13 | 114378 | 114390 | *rpl32-trnP(GGG)* |
| 99 | p1 | spacer | SSC | A | 13 | 114717 | 114729 | *trnP(GGG)-trnL(UAG)* |
| 100 | p1 | spacer | SSC | A | 11 | 114877 | 114887 | *trnL(UAG)-ccsA* |
| 101 | p1 | spacer | SSC | T | 12 | 114956 | 114967 | *trnL(UAG)-ccsA* |
| 102 | p1 | spacer | SSC | A | 11 | 118109 | 118119 | *ndhD-psaC* |
| 103 | p1 | spacer | SSC | T | 12 | 118124 | 118135 | *ndhD-psaC* |
| 104 | p1 | spacer | SSC | G | 13 | 118671 | 118683 | *psaC-ndhE* |
| 105 | p2 | spacer | SSC | TA | 14 | 120486 | 120499 | *ndhI-ndhA* |
| 106 | p1 | spacer | SSC | A | 13 | 120503 | 120515 | *ndhI-ndhA* |
| 107 | p2 | spacer | SSC | AT | 20 | 120515 | 120534 | *ndhI-ndhA* |
| 108 | p1 | spacer | SSC | A | 10 | 120579 | 120588 | *ndhI-ndhA* |
| 109 | p1 | intron | SSC | T | 10 | 121499 | 121508 | *ndhA* |
| 110 | p1 | spacer | SSC | A | 14 | 124291 | 124304 | *rps15-ycf1* |
| 111 | p4 | CDS | SSC | ACCA | 12 | 129075 | 129086 | *ycf1* |
| 112 | p1 | spacer | SSC | C | 13 | 129616 | 129628 | *ycf1-chlN* |
| 113 | p1 | spacer | IRA | C | 15 | 132548 | 132562 | *chlL-trnN(GUU)* |
| 114 | p1 | spacer | IRA | T | 23 | 133339 | 133361 | *trnN(GUU)-ycf2* |
| 115 | p4 | CDS | IRA | TTTC | 12 | 134759 | 134770 | *ycf2* |
| 116 | p1 | spacer | IRA | T | 12 | 139936 | 139947 | *ycf2-trnH(GUG)* |
| 117 | p1 | spacer | IRA | A | 11 | 143895 | 143905 | *rps12-rrn16* |
| 118 | p4 | spacer | IRA | AAAG | 12 | 145006 | 145017 | *rps12-rrn16* |
| 119 | p1 | spacer | IRA | G | 11 | 145539 | 145549 | *rps12-rrn16* |
| 120 | p1 | spacer | IRA | A | 13 | 153065 | 153077 | *rrn5-trnR(ACG)* |
| 121 | p2 | spacer | IRA | AT | 12 | 154513 | 154524 | *trnR(ACG)-trnT(UGU)* |
| 122 | p2 | spacer | IRA | AT | 12 | 154968 | 154979 | *trnT(UGU)* |

Appendix Table S7 List of simple sequence repeats (SSRs) in the chloroplast genomes of *Sphaeropteris brunoniana*

| SSR nr. | SSR type | Locus | Region | SSR motif | size | star | end | Location |
| --- | --- | --- | --- | --- | --- | --- | --- | --- |
| 1 | p1 | spacer | LSC | A | 15 | 3517 | 3531 | *matK-rps16* |
| 2 | p1 | spacer | LSC | T | 10 | 3614 | 3623 | *matK-rps16* |
| 3 | p1 | intron | LSC | T | 10 | 4364 | 4373 | *rps16* |
| 4 | p1 | intron | LSC | A | 11 | 4861 | 4871 | *rps16* |
| 5 | p1 | spacer | LSC | G | 10 | 6893 | 6902 | *chlB-trnQ(UUG)* |
| 6 | p1 | spacer | LSC | A | 12 | 7068 | 7619 | *psbK-psbI* |
| 7 | p2 | spacer | LSC | TA | 12 | 7621 | 7632 | *psbK-psbI* |
| 8 | p1 | spacer | LSC | C | 17 | 8230 | 8246 | *trnS(GCU)-psaM* |
| 9 | p1 | spacer | LSC | A | 11 | 8332 | 8342 | *trnS(GCU)-psaM* |
| 10 | p1 | spacer | LSC | T | 12 | 8359 | 8370 | *trnS(GCU)-psaM* |
| 11 | p1 | intron | LSC | T | 12 | 9637 | 9648 | *trnG(UCC)* |
| 12 | p2 | intron | LSC | TC | 14 | 12639 | 12652 | *atpF* |
| 13 | p1 | spacer | LSC | C | 16 | 13302 | 13317 | *atpF-atpH* |
| 14 | p1 | spacer | LSC | T | 13 | 15171 | 15183 | *atpI-rps2* |
| 15 | p1 | spacer | LSC | A | 10 | 23386 | 23395 | *rpoC1-rpoB* |
| 16 | p1 | spacer | LSC | T | 12 | 27644 | 27655 | *rpoB-trnD(GUC)* |
| 17 | p1 | spacer | LSC | A | 11 | 28256 | 28266 | *trnE(UUC)-psbM* |
| 18 | p1 | spacer | LSC | T | 22 | 28291 | 28312 | *trnE(UUC)-psbM* |
| 19 | p1 | spacer | LSC | A | 14 | 29431 | 29444 | *ycf66* |
| 20 | p1 | spacer | LSC | G | 14 | 29522 | 29535 | *ycf66* |
| 21 | p1 | spacer | LSC | T | 13 | 30206 | 30218 | *petN-trnC(GCA)* |
| 22 | p1 | spacer | LSC | T | 11 | 30804 | 30814 | *trnC(GCA)-trnG(GCC)* |
| 23 | p4 | spacer | LSC | TATC | 16 | 33672 | 33687 | *trnS(UGA)-psbC* |
| 24 | p4 | spacer | LSC | ATAG | 16 | 33691 | 33706 | *trnS(UGA)-psbC* |
| 25 | p1 | spacer | LSC | T | 11 | 36882 | 36892 | *psbD-trnT(GGU)* |
| 26 | p1 | spacer | LSC | A | 10 | 36920 | 36929 | *psbD-trnT(GGU)* |
| 27 | p1 | spacer | LSC | T | 19 | 37334 | 37352 | *psbD-trnT(GGU)* |
| 28 | p1 | spacer | LSC | T | 21 | 37588 | 37608 | *psbD-trnT(GGU)* |
| 29 | p4 | spacer | LSC | ATAG | 12 | 38356 | 38367 | *trnfM(CAU)-rps14* |
| 30 | p1 | spacer | LSC | T | 10 | 38371 | 38380 | *trnfM(CAU)-rps14* |
| 31 | p1 | intron | LSC | T | 21 | 44159 | 44179 | *ycf3* |
| 32 | p1 | spacer | LSC | T | 23 | 45660 | 45682 | *ycf3-trnS(GGA)* |
| 33 | p1 | spacer | LSC | T | 18 | 45968 | 45985 | *ycf3-trnS(GGA)* |
| 34 | p1 | spacer | LSC | T | 13 | 46269 | 46281 | *trnS(GGA)-rps4* |
| 35 | p1 | intron | LSC | A | 11 | 48282 | 48292 | *trnL(CAA)* |
| 36 | p2 | spacer | LSC | TA | 12 | 48647 | 48658 | *trnL(CAA)-trnF(GAA)* |
| 37 | p2 | spacer | LSC | TA | 24 | 48661 | 48684 | *trnL(CAA)-trnF(GAA)* |
| 38 | p1 | spacer | LSC | T | 12 | 51179 | 51190 | *ndhC-trnV(UAC)* |
| 39 | p1 | intron | LSC | T | 10 | 51514 | 51523 | *trnV(UAC)* |
| 40 | p1 | spacer | LSC | A | 16 | 52031 | 52046 | *trnV(UAC)-trnM(CAU)* |
| 41 | p1 | spacer | LSC | T | 10 | 54498 | 54507 | *atpB-rbcL* |
| 42 | p1 | spacer | LSC | T | 12 | 56441 | 56452 | *rbcL-trnR(UCG)* |
| 43 | p1 | spacer | LSC | T | 14 | 56859 | 56872 | *trnR(UCG)-accD* |
| 44 | p1 | spacer | LSC | T | 10 | 58386 | 58395 | *accD-psaI* |
| 45 | p2 | spacer | LSC | AT | 16 | 62683 | 62698 | *petA-psbJ* |
| 46 | p1 | spacer | LSC | T | 13 | 63021 | 63033 | *petA-psbJ* |
| 47 | p1 | spacer | LSC | T | 16 | 64669 | 64684 | *psbE-petL* |
| 48 | p1 | spacer | LSC | A | 12 | 65041 | 65052 | *petL-petG* |
| 49 | p1 | spacer | LSC | C | 10 | 66553 | 66562 | *psaJ-rpl33* |
| 50 | p1 | spacer | LSC | A | 13 | 67731 | 67743 | *rpl20-rps12* |
| 51 | p1 | spacer | LSC | T | 13 | 67974 | 67986 | *rpl20-rps12* |
| 52 | p1 | spacer | LSC | A | 15 | 68134 | 68148 | *rpl20-rps12* |
| 53 | p1 | spacer | LSC | T | 11 | 68406 | 68416 | *rpl20-rps12* |
| 54 | p4 | spacer | LSC | TATC | 12 | 69106 | 69117 | *rps12-clpP* |
| 55 | p2 | intron | LSC | TA | 12 | 70495 | 70506 | *clpP* |
| 56 | p1 | intron | LSC | C | 14 | 70536 | 70549 | *clpP* |
| 57 | p1 | spacer | LSC | A | 12 | 71504 | 71515 | *clpP-psbB* |
| 58 | p1 | spacer | LSC | T | 11 | 71597 | 71607 | *clpP-psbB* |
| 59 | p1 | spacer | LSC | A | 18 | 73387 | 73404 | *psbB-psbT* |
| 60 | p1 | intron | LSC | T | 10 | 74735 | 74744 | *petB* |
| 61 | p1 | spacer | LSC | T | 10 | 77271 | 77280 | *petD-ropA* |
| 62 | p1 | spacer | LSC | T | 11 | 77328 | 77338 | *petD-ropA* |
| 63 | p1 | spacer | LSC | T | 14 | 80050 | 80063 | *rps8-rpl14* |
| 64 | p2 | spacer | LSC | AT | 14 | 80083 | 80096 | *rps8-rpl14* |
| 65 | p2 | spacer | LSC | AT | 18 | 80177 | 80194 | *rps8-rpl14* |
| 66 | p1 | spacer | LSC | A | 13 | 81030 | 81042 | *rpl14-rpl16* |
| 67 | p1 | intron | LSC | T | 11 | 81765 | 81775 | *rpl16* |
| 68 | p1 | intron | LSC | T | 11 | 82107 | 82117 | *rpl16* |
| 69 | p2 | spacer | LSC | AT | 14 | 82324 | 82337 | *rpl16-rps3* |
| 70 | p1 | intron | LSC | A | 10 | 84458 | 84467 | *rpl2* |
| 71 | p1 | intron | LSC | A | 10 | 84570 | 84549 | *rpl2* |
| 72 | p2 | intron | LSC | AT | 12 | 84561 | 84572 | *rpl2* |
| 73 | p1 | intron | LSC | T | 14 | 84852 | 84865 | *rpl2* |
| 74 | p1 | spacer | LSC | T | 13 | 85845 | 85857 | *rpl23-trnI(CAU)* |
| 75 | p1 | spacer | LSC | T | 13 | 85991 | 86003 | *rpl23-trnI(CAU)* |
| 76 | p4 | rRNA gene | IRA | CTAC | 12 | 91186 | 91197 | *rrn23* |
| 77 | p2 | intron | IRA | AG | 12 | 94186 | 94197 | *trnI(CAU)* |
| 78 | p4 | spacer | IRA | TCTT | 12 | 97474 | 97485 | *rrn16-rps12* |
| 79 | p1 | spacer | IRA | T | 14 | 98567 | 98580 | *rrn16-rps12* |
| 80 | p1 | spacer | IRA | A | 12 | 102544 | 102555 | *trnH(GUG)-ycf2* |
| 81 | p4 | CDS | IRA | AGAA | 12 | 107744 | 107755 | *ycf2* |
| 82 | p1 | spacer | IRA | A | 15 | 109158 | 109172 | *ycf2-trnN(GUU)* |
| 83 | p1 | spacer | SSC | T | 11 | 110207 | 110217 | *trnN(GUU)-ndhF* |
| 84 | p1 | spacer | SSC | A | 14 | 113545 | 113558 | *rpl32-trnP(GGG)* |
| 85 | p1 | spacer | SSC | A | 11 | 113825 | 113835 | *rpl32-trnP(GGG)* |
| 86 | p1 | spacer | SSC | T | 15 | 113869 | 113883 | *rpl32-trnP(GGG)* |
| 87 | p1 | spacer | SSC | A | 13 | 114374 | 114386 | *trnL(UAG)-ccsA* |
| 88 | p2 | spacer | SSC | AT | 20 | 114464 | 114483 | *trnL(UAG)-ccsA* |
| 89 | p1 | spacer | SSC | A | 13 | 115715 | 115727 | *ccsA-ndhD* |
| 90 | p1 | spacer | SSC | A | 14 | 115972 | 115985 | *ccsA-ndhD* |
| 91 | p1 | spacer | SSC | A | 14 | 117633 | 117646 | *ndhD-psaC* |
| 92 | p2 | spacer | SSC | CT | 12 | 119317 | 119328 | *ndhG-ndhI* |
| 93 | p2 | intron | SSC | AT | 16 | 121036 | 121051 | *ndhA* |
| 94 | p1 | intron | SSC | T | 11 | 121544 | 121554 | *ndhA* |
| 95 | p1 | intron | SSC | A | 11 | 123840 | 123850 | *rps15-ycf1* |
| 96 | p1 | spacer | IRB | T | 15 | 133684 | 133698 | *trnN(GUU)-ycf2* |
| 97 | p4 | CDS | IRB | TTTC | 12 | 135100 | 135111 | *ycf2* |
| 98 | p1 | spacer | IRB | T | 12 | 140301 | 140312 | *ycf2-trnH(GUG)* |
| 99 | p1 | spacer | IRB | A | 14 | 144276 | 144289 | *rps12-rrn16* |
| 100 | p4 | spacer | IRB | AAAG | 12 | 145370 | 145381 | *rps12-rrn16* |
| 101 | p2 | intron | IRB | TC | 12 | 148658 | 148669 | *trnI(GUA)* |
| 102 | p4 | rRNA gene | IRB | AGGT | 12 | 151657 | 151668 | *rrn23* |

Appendix Table S8 List of simple sequence repeats (SSRs) in the chloroplast genomes of *Sphaeropteris lepifera*

| SSR nr. | SSR type | Locus | Region | SSR motif | size | star | end | Location |
| --- | --- | --- | --- | --- | --- | --- | --- | --- |
| 1 | p1 | spacer | LSC | A | 13 | 3514 | 3526 | *matK-rps16* |
| 2 | p1 | spacer | LSC | T | 10 | 3613 | 3622 | *matK-rps16* |
| 3 | p2 | spacer | LSC | TA | 16 | 7615 | 7630 | *psbK-psbI* |
| 4 | p1 | spacer | LSC | C | 14 | 8229 | 8242 | *trnS(GCU)-psaM* |
| 5 | p1 | spacer | LSC | A | 22 | 8332 | 8353 | *trnS(GCU)-psaM* |
| 6 | p1 | spacer | LSC | T | 11 | 8370 | 8380 | *trnS(GCU)-psaM* |
| 7 | p1 | intron | LSC | A | 10 | 9507 | 9516 | *ycf12* |
| 8 | p1 | intron | LSC | T | 12 | 9648 | 9659 | *ycf12* |
| 9 | p1 | spacer | LSC | C | 38 | 13308 | 13345 | *atpF-atpH* |
| 10 | p1 | spacer | LSC | T | 12 | 15193 | 15204 | *atpI-rps2* |
| 11 | p1 | intron | LSC | C | 10 | 22421 | 22430 | *rpoC1* |
| 12 | p1 | spacer | LSC | A | 12 | 23409 | 23420 | *rpoC1-rpoB* |
| 13 | p1 | spacer | LSC | T | 14 | 27774 | 27787 | *rpoB-trnD(GUC)* |
| 14 | p1 | spacer | LSC | A | 14 | 28387 | 28400 | *trnE(UUC)-psbM* |
| 15 | p1 | spacer | LSC | T | 21 | 28425 | 28445 | *trnE(UUC)-psbM* |
| 16 | p1 | spacer | LSC | A | 12 | 29887 | 29898 | *ycf66* |
| 17 | p1 | spacer | LSC | T | 10 | 30334 | 30343 | *petN-trnC(GCA)* |
| 18 | p1 | spacer | LSC | A | 15 | 30525 | 30539 | *petN-trnC(GCA)* |
| 19 | p1 | spacer | LSC | T | 10 | 30938 | 30947 | *trnC(GCA)-trnG(GCC)* |
| 20 | p1 | spacer | LSC | T | 12 | 32361 | 32372 | *trnG(GCC)-psbZ* |
| 21 | p1 | spacer | LSC | T | 10 | 33284 | 33293 | *trnS(UGA)-psbC* |
| 22 | p4 | spacer | LSC | TATC | 16 | 33813 | 33828 | *trnS(UGA)-psbC* |
| 23 | p4 | spacer | LSC | ATAG | 12 | 33832 | 33843 | *trnS(UGA)-psbC* |
| 24 | p1 | spacer | LSC | T | 16 | 37027 | 37042 | *psbD-trnT(GGU)* |
| 25 | p1 | spacer | LSC | A | 13 | 37070 | 37082 | *psbD-trnT(GGU)* |
| 26 | p1 | spacer | LSC | T | 19 | 37491 | 37509 | *psbD-trnT(GGU)* |
| 27 | p1 | spacer | LSC | T | 12 | 37753 | 37764 | *psbD-trnT(GGU)* |
| 28 | p2 | spacer | LSC | TA | 16 | 38517 | 38532 | *trnfM-rps14* |
| 29 | p1 | spacer | LSC | T | 10 | 38541 | 38550 | *trnfM-rps14* |
| 30 | p1 | intron | LSC | T | 13 | 44335 | 44347 | *ycf3* |
| 31 | p1 | intron | LSC | C | 10 | 45444 | 45453 | *ycf3* |
| 32 | p1 | spacer | LSC | T | 12 | 45838 | 45849 | *ycf3-trnS(GGA)* |
| 33 | p1 | spacer | LSC | T | 12 | 46134 | 46145 | *ycf3-trnS(GGA)* |
| 34 | p1 | spacer | LSC | T | 11 | 46434 | 46444 | *trnS(GGA)-rps4* |
| 35 | p1 | intron | LSC | A | 10 | 48446 | 48455 | *trnL(CAA)* |
| 36 | p2 | spacer | LSC | TA | 14 | 48820 | 48833 | *trnL(CAA)-trnF(GAA)* |
| 37 | p1 | spacer | LSC | T | 10 | 51310 | 51319 | *ndhC-trnV(UAC)* |
| 38 | p1 | spacer | LSC | A | 14 | 52158 | 52171 | *trnV(UAC)-trnM(CAU)* |
| 39 | p1 | spacer | LSC | T | 11 | 54626 | 54636 | *atpB-rbcL* |
| 40 | p1 | spacer | LSC | T | 13 | 56570 | 56582 | *rbcL-trnR(UCG)* |
| 41 | p1 | spacer | LSC | T | 13 | 56989 | 57001 | *trnR(UCG)-accD* |
| 42 | p1 | spacer | LSC | A | 10 | 58098 | 58107 | *accD_psaI* |
| 43 | p1 | spacer | LSC | T | 12 | 58522 | 58533 | *accD_psaI* |
| 44 | p1 | spacer | LSC | A | 11 | 59707 | 59717 | *ycf4-cemA* |
| 45 | p1 | spacer | LSC | A | 10 | 62694 | 62703 | *petA-psbJ* |
| 46 | p1 | spacer | LSC | T | 14 | 63180 | 63193 | *petA-psbJ* |
| 47 | p1 | spacer | LSC | T | 10 | 64840 | 64849 | *psbE-petL* |
| 48 | p1 | spacer | LSC | A | 10 | 65211 | 65220 | *petL-petG* |
| 49 | p1 | spacer | LSC | C | 12 | 66700 | 66711 | *psaJ-rpl33* |
| 50 | p1 | spacer | LSC | A | 12 | 67888 | 67899 | *rpl20-rps12* |
| 51 | p1 | spacer | LSC | T | 13 | 68130 | 68142 | *rpl20-rps12* |
| 52 | p1 | spacer | LSC | A | 10 | 68290 | 68299 | *rpl20-rps12* |
| 53 | p1 | spacer | LSC | T | 11 | 68557 | 68567 | *rpl20-rps12* |
| 54 | p4 | spacer | LSC | TTAT | 12 | 69379 | 69390 | *rps12-clpP* |
| 55 | p1 | intron | LSC | C | 10 | 70697 | 70706 | *clpP* |
| 56 | p1 | spacer | LSC | A | 13 | 71660 | 71672 | *clpP-psbB* |
| 57 | p1 | spacer | LSC | T | 10 | 71754 | 71763 | *clpP-psbB* |
| 58 | p1 | spacer | LSC | A | 13 | 73543 | 73555 | *psbB-psbT* |
| 59 | p1 | spacer | LSC | A | 10 | 74268 | 74277 | *psbH-petB* |
| 60 | p1 | spacer | LSC | T | 13 | 77480 | 77492 | *petD-rpoA* |
| 61 | p2 | spacer | LSC | AT | 20 | 80238 | 80257 | *rps8-rpl14* |
| 62 | p2 | spacer | LSC | AT | 16 | 80338 | 80353 | *rps8-rpl14* |
| 63 | p1 | spacer | LSC | A | 13 | 81186 | 81198 | *rpl14-rpl16* |
| 64 | p1 | intron | LSC | T | 12 | 81919 | 81930 | *rpl16* |
| 65 | p1 | intron | LSC | T | 12 | 82262 | 82273 | *rpl16* |
| 66 | p1 | spacer | LSC | T | 11 | 82483 | 82493 | *rpl16-rps3* |
| 67 | p1 | intron | LSC | T | 15 | 84995 | 85009 | *rpl2* |
| 68 | p1 | spacer | LSC | A | 11 | 85683 | 85693 | *rpl2-rpl23* |
| 69 | p1 | spacer | LSC | T | 12 | 85991 | 86002 | *rpl23-trnI(CAU)* |
| 70 | p1 | spacer | LSC | T | 16 | 86136 | 86151 | *rpl23-trnI(CAU)* |
| 71 | p4 | rRNA gene | IRB | CTAC | 12 | 91392 | 91403 | *rrn23* |
| 72 | p2 | intron | IRB | AG | 14 | 94391 | 94404 | *trnI(GAU)* |
| 73 | p4 | spacer | IRB | TCTT | 12 | 97681 | 97692 | *rrn16-rps12* |
| 74 | p1 | spacer | IRB | T | 13 | 98774 | 98786 | *rrn16-rps12* |
| 75 | p1 | spacer | IRB | A | 12 | 102750 | 102761 | *trnH(GUG)-ycf2* |
| 76 | p4 | CDS | IRB | TTTA | 12 | 104706 | 104717 | *ycf2* |
| 77 | p4 | CDS | IRB | AGAA | 12 | 107950 | 107961 | *ycf2* |
| 78 | p1 | spacer | IRB | A | 15 | 109364 | 109378 | *ycf2-trnN(GUU)* |
| 79 | p1 | spacer | SSC | T | 13 | 110416 | 110428 | *ycf2-trnN(GUU)* |
| 80 | p1 | spacer | SSC | A | 11 | 112695 | 112705 | *ndhF-rpl21* |
| 81 | p2 | spacer | SSC | AT | 18 | 113419 | 113436 | *rpl21-rpl32* |
| 82 | p1 | spacer | SSC | A | 11 | 113761 | 113771 | *rpl32-trnP(GGG)* |
| 83 | p1 | spacer | SSC | T | 13 | 114080 | 114092 | *rpl32-trnP(GGG)* |
| 84 | p1 | spacer | SSC | T | 12 | 119615 | 119626 | *trnP(GGG)-trnL(UAG)* |
| 85 | p1 | spacer | SSC | A | 10 | 119831 | 119840 | *trnL(UAG)-ccsA* |
| 86 | p2 | spacer | SSC | AT | 16 | 119920 | 119935 | *trnL(UAG)-ccsA* |
| 87 | p1 | spacer | SSC | A | 11 | 121168 | 121178 | *ccsA-ndhD* |
| 88 | p1 | spacer | SSC | A | 14 | 121427 | 121440 | *ccsA-ndhD* |
| 89 | p1 | spacer | SSC | A | 16 | 123088 | 123103 | *ndhD-psaC* |
| 90 | p4 | spacer | SSC | TTAA | 12 | 123104 | 123155 | *ndhD-psaC* |
| 91 | p2 | spacer | SSC | CT | 12 | 124783 | 124794 | *ndhG-ndhI* |
| 92 | p2 | intron | SSC | TA | 12 | 126492 | 126503 | *ndhA* |
| 93 | p1 | intron | SSC | T | 13 | 127011 | 127023 | *ndhA* |
| 94 | p1 | spacer | SSC | A | 10 | 129309 | 129318 | *rps15-ycf1* |
| 95 | p1 | spacer | SSC | A | 10 | 129333 | 129342 | *rps15-ycf1* |
| 96 | p1 | spacer | IRA | T | 15 | 139188 | 139202 | *trnN(GUU)-ycf2* |
| 97 | p4 | CDS | IRA | TTTC | 12 | 140604 | 140615 | *ycf2* |
| 98 | p4 | CDS | IRA | TAAA | 12 | 143849 | 143860 | *ycf2* |
| 99 | p1 | spacer | IRA | T | 12 | 145805 | 145816 | *ycf2-trnH(GUG)* |
| 100 | p1 | spacer | IRA | A | 13 | 149780 | 149792 | *rps12-rrn16* |
| 101 | p4 | spacer | IRA | AAAG | 12 | 150873 | 150884 | *rps12-rrn16* |
| 102 | p2 | intron | IRA | TC | 14 | 154161 | 154174 | *trnI(GAU)* |
| 103 | p4 | rRNA gene | IRA | AGGT | 12 | 157161 | 157172 | *rrn23* |

Appendix Table S9 The number, relative abundance, relative density, and GC content of SSR motifs in the chloroplast genomes of eight Cyatheaceae species

| motif type | Characteristics of SSR | *G. denticulata* | *G. podophylla* | *G. gigantea* | *G.metteniana* | *A. costularis* | *A. spinulosa* | *S. brunoniana* | *S. lepifera* |
| --- | --- | --- | --- | --- | --- | --- | --- | --- | --- |
| A | No. of SSRs | 13 | 10 | 10 | 11 | 31 | 31 | 28 | 32 |
|  | Relative abundance (No./Kb) | 0.084 | 0.060 | 0.062 | 0.068 | 0.198 | 0.198 | 0.179 | 0.197 |
|  | Relative density (bp/Kb) | 0.883 | 0.614 | 0.798 | 0.860 | 2.374 | 2.496 | 2.266 | 2.410 |
| C | No. of SSRs | 8 | 8 | 8 | 6 | 10 | 11 | 4 | 6 |
|  | Relative abundance (No./Kb) | 0.052 | 0.048 | 0.049 | 0.037 | 0.064 | 0.070 | 0.026 | 0.037 |
|  | Relative density (bp/Kb) | 0.565 | 0.554 | 0.637 | 0.421 | 1.040 | 0.855 | 0.364 | 0.579 |
| G | No. of SSRs | 6 | 7 | 7 | 6 | 8 | 8 | 2 | 0 |
|  | Relative abundance (No./Kb) | 0.039 | 0.042 | 0.043 | 0.037 | 0.051 | 0.051 | 0.013 | 0 |
|  | Relative density (bp/Kb) | 0.487 | 0.542 | 0.619 | 0.446 | 0.645 | 0.613 | 0.153 | 0 |
| T | No. of SSRs | 13 | 22 | 16 | 17 | 46 | 46 | 43 | 42 |
|  | Relative abundance (No./Kb) | 0.084 | 0.132 | 0.099 | 0.105 | 0.294 | 0.294 | 0.274 | 0.259 |
|  | Relative density (bp/Kb) | 0.993 | 1.450 | 1.076 | 1.157 | 3.606 | 3.632 | 3.626 | 3.212 |
| AG | No. of SSRs | 0 | 0 | 1 | 1 | 0 | 0 | 1 | 1 |
|  | Relative abundance (No./Kb) | 0 | 0 | 0.006 | 0.006 | 0 | 0 | 0.006 | 0.006 |
|  | Relative density (bp/Kb) | 0 | 0 | 0.148 | 0.099 | 0 | 0 | 0.077 | 0.086 |
| AT | No. of SSRs | 5 | 6 | 6 | 6 | 11 | 11 | 7 | 4 |
|  | Relative abundance (No./Kb) | 0.032 | 0.036 | 0.037 | 0.037 | 0.070 | 0.070 | 0.045 | 0.025 |
|  | Relative density (bp/Kb) | 0.454 | 0.518 | 0.495 | 0.520 | 1.111 | 1.111 | 0.702 | 0.432 |
| CT | No. of SSRs | 1 | 0 | 0 | 0 | 0 | 0 | 1 | 1 |
|  | Relative abundance (No./Kb) | 0.006 | 0 | 0 | 0 | 0 | 0 | 0.006 | 0.006 |
|  | Relative density (bp/Kb) | 0.078 | 0 | 0 | 0 | 0 | 0 | 0.077 | 0.074 |
| TA | No. of SSRs | 2 | 3 | 3 | 3 | 3 | 3 | 4 | 4 |
|  | Relative abundance (No./Kb) | 0.013 | 0.018 | 0.019 | 0.019 | 0.019 | 0.019 | 0.026 | 0.025 |
|  | Relative density (bp/Kb) | 0.156 | 0.313 | 0.297 | 0.297 | 0.243 | 0.243 | 0.383 | 0.358 |
| TC | No. of SSRs | 0 | 0 | 0 | 0 | 0 | 0 | 2 | 1 |
|  | Relative abundance (No./Kb) | 0 | 0 | 0 | 0 | 0 | 0 | 0.013 | 0.006 |
|  | Relative density (bp/Kb) | 0 | 0 | 0 | 0 | 0 | 0 | 0.166 | 0.086 |
| AAG | No. of SSRs | 0 | 0 | 0 | 0 | 1 | 1 | 0 | 0 |
|  | Relative abundance (No./Kb) | 0 | 0 | 0 | 0 | 0.006 | 0.006 | 0 | 0 |
|  | Relative density (bp/Kb) | 0 | 0 | 0 | 0 | 0.077 | 0.077 | 0 | 0 |
| AAT | No. of SSRs | 1 | 1 | 1 | 1 | 0 | 0 | 0 | 0 |
|  | Relative abundance (No./Kb) | 0.006 | 0.006 | 0.006 | 0.006 | 0 | 0 | 0 | 0 |
|  | Relative density (bp/Kb) | 0.078 | 0.072 | 0.074 | 0.074 | 0 | 0 | 0 | 0 |
| TAT | No. of SSRs | 1 | 0 | 1 | 1 | 0 | 0 | 0 | 0 |
|  | Relative abundance (No./Kb) | 0.006 | 0 | 0.006 | 0.006 | 0 | 0 | 0 | 0 |
|  | Relative density (bp/Kb) | 0.078 | 0 | 0.074 | 0.074 | 0 | 0 | 0 | 0 |
| AAAG | No. of SSRs | 1 | 0 | 1 | 1 | 1 | 1 | 1 | 1 |
|  | Relative abundance (No./Kb) | 0.006 | 0 | 0.006 | 0.006 | 0.006 | 0.006 | 0.006 | 0.006 |
|  | Relative density (bp/Kb) | 0.078 | 0 | 0.074 | 0.074 | 0.077 | 0.077 | 0.077 | 0.074 |
| AAAT | No. of SSRs | 0.000 | 1 | 1 | 1 | 0 | 0 | 0 | 0 |
|  | Relative abundance (No./Kb) | 0.000 | 0.006 | 0.006 | 0.006 | 0 | 0 | 0 | 0 |
|  | Relative density (bp/Kb) | 0.000 | 0.072 | 0.099 | 0.099 | 0 | 0 | 0 | 0 |
| AATT | No. of SSRs | 2 | 2 | 1 | 2 | 0 | 0 | 0 | 0 |
|  | Relative abundance (No./Kb) | 0.013 | 0.012 | 0.006 | 0.012 | 0 | 0 | 0 | 0 |
|  | Relative density (bp/Kb) | 0.156 | 0.144 | 0.074 | 0.149 | 0 | 0 | 0 | 0 |
| ACCA | No. of SSRs | 0 | 0 | 0 | 0 | 1 | 1 | 0 | 0 |
|  | Relative abundance (No./Kb) | 0 | 0 | 0 | 0 | 0.006 | 0.006 | 0 | 0 |
|  | Relative density (bp/Kb) | 0 | 0 | 0 | 0 | 0.077 | 0.077 | 0 | 0 |
| AGAA | No. of SSRs | 0 | 0 | 0 | 0 | 1 | 1 | 1 | 1 |
|  | Relative abundance (No./Kb) | 0 | 0 | 0 | 0 | 0.006 | 0.006 | 0.006 | 0.006 |
|  | Relative density (bp/Kb) | 0 | 0 | 0 | 0 | 0.077 | 0.077 | 0.077 | 0.074 |
| AGGT | No. of SSRs | 0 | 0 | 0 | 0 | 0 | 0 | 1 | 1 |
|  | Relative abundance (No./Kb) | 0 | 0 | 0 | 0 | 0 | 0 | 0.006 | 0.006 |
|  | Relative density (bp/Kb) | 0 | 0 | 0 | 0 | 0 | 0 | 0.077 | 0.074 |
| ATAG | No. of SSRs | 2 | 2 | 2 | 2 | 1 | 1 | 2 | 1 |
|  | Relative abundance (No./Kb) | 0.013 | 0.012 | 0.012 | 0.012 | 0.006 | 0.006 | 0.013 | 0.006 |
|  | Relative density (bp/Kb) | 0.156 | 0.144 | 0.148 | 0.149 | 0.077 | 0.077 | 0.179 | 0.074 |
| ATCT | No. of SSRs | 0 | 0 | 0 | 0 | 1 | 1 | 0 | 0 |
|  | Relative abundance (No./Kb) | 0 | 0 | 0 | 0 | 0.006 | 0.006 | 0 | 0 |
|  | Relative density (bp/Kb) | 0 | 0 | 0 | 0 | 0.102 | 0.102 | 0 | 0 |
| CCTC | No. of SSRs | 0 | 0 | 0 | 0 | 1 | 1 | 0 | 0 |
|  | Relative abundance (No./Kb) | 0 | 0 | 0 | 0 | 0.006 | 0.006 | 0 | 0 |
|  | Relative density (bp/Kb) | 0 | 0 | 0 | 0 | 0.077 | 0.077 | 0 | 0 |
| CTAC | No. of SSRs | 0 | 0 | 0 | 0 | 0 | 0 | 1 | 1 |
|  | Relative abundance (No./Kb) | 0 | 0 | 0 | 0 | 0 | 0 | 0.006 | 0.006 |
|  | Relative density (bp/Kb) | 0 | 0 | 0 | 0 | 0 | 0 | 0.077 | 0.074 |
| GAAA | No. of SSRs | 0 | 0 | 1 | 1 | 0 | 0 | 0 | 0 |
|  | Relative abundance (No./Kb) | 0 | 0 | 0.006 | 0.006 | 0 | 0 | 0 | 0 |
|  | Relative density (bp/Kb) | 0 | 0 | 0.074 | 0.074 | 0 | 0 | 0 | 0 |
| GATA | No. of SSRs | 1 | 1 | 1 | 1 | 1 | 1 | 0 | 0 |
|  | Relative abundance (No./Kb) | 0.006 | 0.006 | 0.006 | 0.006 | 0.006 | 0.006 | 0 | 0 |
|  | Relative density (bp/Kb) | 0.078 | 0.072 | 0.074 | 0.074 | 0.077 | 0.077 | 0 | 0 |
| TAAA | No. of SSRs | 0 | 0 | 0 | 0 | 0 | 0 | 0 | 1 |
|  | Relative abundance (No./Kb) | 0 | 0 | 0 | 0 | 0 | 0 | 0 | 0.006 |
|  | Relative density (bp/Kb) | 0 | 0 | 0 | 0 | 0 | 0 | 0 | 0.074 |
| TATC | No. of SSRs | 1 | 1 | 1 | 1 | 1 | 1 | 2 | 1 |
|  | Relative abundance (No./Kb) | 0.006 | 0.006 | 0.006 | 0.006 | 0.006 | 0.006 | 0.013 | 0.006 |
|  | Relative density (bp/Kb) | 0.104 | 0.096 | 0.074 | 0.074 | 0.077 | 0.077 | 0.179 | 0.099 |
| TATT | No. of SSRs | 1 | 1 | 1 | 1 | 1 | 1 | 0 | 0 |
|  | Relative abundance (No./Kb) | 0.006 | 0.006 | 0.006 | 0.006 | 0.006 | 0.006 | 0 | 0 |
|  | Relative density (bp/Kb) | 0.078 | 0.072 | 0.074 | 0.074 | 0.077 | 0.077 | 0 | 0 |
| TCTT | No. of SSRs | 1 | 0 | 1 | 1 | 1 | 1 | 1 | 1 |
|  | Relative abundance (No./Kb) | 0.006 | 0 | 0.006 | 0.006 | 0.006 | 0.006 | 0.006 | 0.006 |
|  | Relative density (bp/Kb) | 0.078 | 0 | 0.074 | 0.074 | 0.077 | 0.077 | 0.077 | 0.074 |
| TGAT | No. of SSRs | 1 | 1 | 1 | 1 | 0 | 0 | 0 | 0 |
|  | Relative abundance (No./Kb) | 0.006 | 0.006 | 0.006 | 0.006 | 0 | 0 | 0 | 0 |
|  | Relative density (bp/Kb) | 0.078 | 0.072 | 0.074 | 0.074 | 0 | 0 | 0 | 0 |
| TTAA | No. of SSRs | 0 | 0 | 0 | 0 | 0 | 0 | 0 | 1 |
|  | Relative abundance (No./Kb) | 0 | 0 | 0 | 0 | 0 | 0 | 0 | 0.006 |
|  | Relative density (bp/Kb) | 0 | 0 | 0 | 0 | 0 | 0 | 0 | 0.074 |
| TTAT | No. of SSRs | 0 | 0 | 0 | 0 | 0 | 0 | 0 | 1 |
|  | Relative abundance (No./Kb) | 0 | 0 | 0 | 0 | 0 | 0 | 0 | 0.006 |
|  | Relative density (bp/Kb) | 0 | 0 | 0 | 0 | 0 | 0 | 0 | 0.074 |
| TTTA | No. of SSRs | 0 | 0 | 0 | 0 | 0 | 0 | 0 | 1 |
|  | Relative abundance (No./Kb) | 0 | 0 | 0 | 0 | 0 | 0 | 0 | 0.006 |
|  | Relative density (bp/Kb) | 0 | 0 | 0 | 0 | 0 | 0 | 0 | 0.074 |
| TTTC | No. of SSRs | 0 | 0 | 0 | 0 | 1 | 1 | 1 | 1 |
|  | Relative abundance (No./Kb) | 0 | 0 | 0 | 0 | 0.006 | 0.006 | 0.006 | 0.006 |
|  | Relative density (bp/Kb) | 0 | 0 | 0 | 0 | 0.077 | 0.077 | 0.077 | 0.074 |
| ATTTT | No. of SSRs | 1 | 0 | 0 | 0 | 0 | 0 | 0 | 0 |
|  | Relative abundance (No./Kb) | 0.006 | 0 | 0 | 0 | 0 | 0 | 0 | 0 |
|  | Relative density (bp/Kb) | 0.097 | 0 | 0 | 0 | 0 | 0 | 0 | 0 |
| CTTTC | No. of SSRs | 0 | 0 | 0 | 1 | 0 | 0 | 0 | 0 |
|  | Relative abundance (No./Kb) | 0 | 0 | 0 | 0.006 | 0 | 0 | 0 | 0 |
|  | Relative density (bp/Kb) | 0 | 0 | 0 | 0.093 | 0 | 0 | 0 | 0 |
| TTCTC | No. of SSRs | 0 | 1 | 0 | 0 | 0 | 0 | 0 | 0 |
|  | Relative abundance (No./Kb) | 0 | 0.006 | 0 | 0 | 0 | 0 | 0 | 0 |
|  | Relative density (bp/Kb) | 0 | 0.090 | 0 | 0 | 0 | 0 | 0 | 0 |

Appendix Table S10 The number, relative abundance, relative density and GC content of different types of motif SSRs among different regions in the chloroplast genomes of eight Cyatheaceae species

| Species | motif type | Characteristics of SSR | IGS | intron | CDS | rRNA gene | LSC | SSC | IRA | IRB |
| --- | --- | --- | --- | --- | --- | --- | --- | --- | --- | --- |
| *G. denticulata* | mononucleotide | No.of SSRs | 30 | 10 | 0 | 0 | 30 | 8 | 1 | 1 |
|  |  | Relative abundance (No./Kb) | 0.63 | 0.66 | 0 | 0 | 0.35 | 0.37 | 0.04 | 0.04 |
|  |  | Relative density (bp/Kb) | 7.03 | 7.82 | 0 | 0 | 3.92 | 4.17 | 0.52 | 0.52 |
|  |  | GC content | 0.40 | 0.27 | 0 | 0 | 0.45 | 0.12 | 0 | 0 |
|  | Dinucleotide | No.of SSRs | 7 | 1 | 0 | 0 | 4 | 2 | 1 | 1 |
|  |  | Relative abundance (No./Kb) | 0.15 | 0.07 | 0 | 0 | 0.05 | 0.09 | 0.04 | 0.04 |
|  |  | Relative density (bp/Kb) | 1.99 | 0.79 | 0 | 0 | 0.63 | 1.30 | 0.52 | 0.52 |
|  |  | GC content | 0 | 0.50 | 0 | 0 | 0.11 | 0 | 0 | 0 |
|  | Trinucleotide | No.of SSRs | 2 | 0 | 0 | 0 | 0 | 2 | 0 | 0 |
|  |  | Relative abundance (No./Kb) | 0.04 | 0 | 0 | 0 | 0 | 0.09 | 0 | 0 |
|  |  | Relative density (bp/Kb) | 0.51 | 0 | 0 | 0 | 0 | 1.11 | 0 | 0 |
|  |  | GC content | 0 | 0 | 0 | 0 | 0 | 0 | 0 | 0 |
|  | Tetranucleotide | No.of SSRs | 9 | 1 | 0 | 0 | 7 | 1 | 1 | 1 |
|  |  | Relative abundance (No./Kb) | 0.19 | 0.07 | 0 | 0 | 0.08 | 0.05 | 0.04 | 0.04 |
|  |  | Relative density (bp/Kb) | 2.37 | 0.79 | 0 | 0 | 1.02 | 0.56 | 0.52 | 0.52 |
|  |  | GC content | 0.20 | 0 | 0 | 0 | 0.18 | 0 | 0.25 | 0.25 |
|  | Pentanucleotide | No.of SSRs | 1 | 0 | 0 | 0 | 1 | 0 | 0 | 0 |
|  |  | Relative abundance (No./Kb) | 0.02 | 0 | 0 | 0 | 0.01 | 0 | 0 | 0 |
|  |  | Relative density (bp/Kb) | 0.32 | 0 | 0 | 0 | 0.17 | 0 | 0 | 0 |
|  |  | GC content | 0 | 0 | 0 | 0 | 0 | 0 | 0 | 0 |
|  | Five nucleotides | Total No. of SSRs | 49 | 12 | 0 | 0 | 42 | 13 | 3 | 3 |
| *G. podophylla* | mononucleotide | No.of SSRs | 38 | 9 | 0 | 0 | 32 | 7 | 4 | 4 |
|  |  | Relative abundance (No./Kb) | 0.66 | 0.59 | 0 | 0 | 0.37 | 0.32 | 0.14 | 0.14 |
|  |  | Relative density (bp/Kb) | 7.33 | 6.78 | 0 | 0 | 4.09 | 3.97 | 1.45 | 1.45 |
|  |  | GC content | 0.41 | 0.11 | 0 | 0 | 0.38 | 0.30 | 0.24 | 0.24 |
|  | Dinucleotide | No.of SSRs | 8 | 1 | 0 | 0 | 4 | 3 | 1 | 1 |
|  |  | Relative abundance (No./Kb) | 0.14 | 0.07 | 0 | 0 | 0.05 | 0.14 | 0.03 | 0.03 |
|  |  | Relative density (bp/Kb) | 2.12 | 1.05 | 0 | 0 | 0.71 | 2.40 | 0.42 | 0.42 |
|  |  | GC content | 0 | 0 | 0 | 0 | 0 | 0 | 0 | 0 |
|  | Trinucleotide | No.of SSRs | 0 | 1 | 0 | 0 | 1 | 0 | 0 | 0 |
|  |  | Relative abundance (No./Kb) | 0 | 0.07 | 0 | 0 | 0.01 | 0 | 0 | 0 |
|  |  | Relative density (bp/Kb) | 0 | 0.79 | 0 | 0 | 0.14 | 0 | 0 | 0 |
|  |  | GC content | 0 | 0 | 0 | 0 | 0 | 0 | 0 | 0 |
|  | Tetranucleotide | No.of SSRs | 8 | 1 | 0 | 0 | 7 | 2 | 0 | 0 |
|  |  | Relative abundance (No./Kb) | 0.14 | 0.07 | 0 | 0 | 0.08 | 0.09 | 0 | 0 |
|  |  | Relative density (bp/Kb) | 1.74 | 0.79 | 0 | 0 | 1.01 | 1.11 | 0 | 0 |
|  |  | GC content | 0.16 | 0 | 0 | 0 | 0.18 | 0 | 0 | 0 |
|  | Pentanucleotide | No.of SSRs | 0 | 1 | 0 | 0 | 1 | 0 | 0 | 0 |
|  |  | Relative abundance (No./Kb) | 0 | 0.07 | 0 | 0 | 0.01 | 0 | 0 | 0 |
|  |  | Relative density (bp/Kb) | 0 | 0.99 | 0 | 0 | 0.17 | 0 | 0 | 0 |
|  |  | GC content | 0 | 0.40 | 0 | 0 | 0.40 | 0 | 0 | 0 |
|  | Five nucleotides | Total No. of SSRs | 54 | 13 | 0 | 0 | 45 | 12 | 5 | 5 |
| *G. gigantea* | mononucleotide | No.of SSRs | 33 | 8 | 0 | 0 | 30 | 7 | 2 | 2 |
|  |  | Relative abundance (No./Kb) | 0.62 | 0.53 | 0 | 0 | 0.32 | 0.32 | 0.08 | 0.08 |
|  |  | Relative density (bp/Kb) | 7.89 | 5.66 | 0 | 0 | 3.52 | 6.13 | 1.01 | 1.01 |
|  |  | GC content | 0.41 | 0.36 | 0 | 0 | 0.43 | 0.47 | 0 | 0 |
|  | Dinucleotide | No.of SSRs | 9 | 1 | 0 | 0 | 5 | 3 | 1 | 1 |
|  |  | Relative abundance (No./Kb) | 0.17 | 0.07 | 0 | 0 | 0.05 | 0.14 | 0.04 | 0.04 |
|  |  | Relative density (bp/Kb) | 2.59 | 0.92 | 0 | 0 | 0.91 | 2.03 | 0.50 | 0.50 |
|  |  | GC content | 0.09 | 0 | 0 | 0 | 0.14 | 0 | 0 | 0 |
|  | Trinucleotide | No.of SSRs | 2 | 0 | 0 | 0 | 0 | 2 | 0 | 0 |
|  |  | Relative abundance (No./Kb) | 0.04 | 0 | 0 | 0 | 0 | 0.09 | 0 | 0 |
|  |  | Relative density (bp/Kb) | 0.45 | 0 | 0 | 0 | 0 | 1.11 | 0 | 0 |
|  |  | GC content | 0 | 0 | 0 | 0 | 0 | 0 | 0 | 0 |
|  | Tetranucleotide | No.of SSRs | 10 | 1 | 0 | 0 | 7 | 2 | 1 | 1 |
|  |  | Relative abundance (No./Kb) | 0.19 | 0.07 | 0 | 0 | 0.08 | 0.09 | 0.04 | 0.04 |
|  |  | Relative density (bp/Kb) | 2.33 | 0.79 | 0 | 0 | 0.91 | 1.29 | 0.50 | 0.50 |
|  |  | GC content | 0.19 | 0 | 0 | 0 | 0.18 | 0.11 | 0.25 | 0.25 |
|  | Pentanucleotide | No.of SSRs | 0 | 0 | 0 | 0 | 0 | 0 | 0 | 0 |
|  |  | Relative abundance (No./Kb) | 0 | 0 | 0 | 0 | 0 | 0 | 0 | 0 |
|  |  | Relative density (bp/Kb) | 0 | 0 | 0 | 0 | 0 | 0 | 0 | 0 |
|  |  | GC content | 0 | 0 | 0 | 0 | 0 | 0 | 0 | 0 |
|  | Five nucleotides | Total No. of SSRs | 54 | 10 | 0 | 0 | 42 | 14 | 4 | 4 |
| *G. metteniana* | mononucleotide | No.of SSRs | 33 | 7 | 0 | 0 | 25 | 9 | 3 | 3 |
|  |  | Relative abundance (No./Kb) | 0.62 | 0.46 | 0 | 0 | 0.27 | 0.42 | 0.13 | 0.13 |
|  |  | Relative density (bp/Kb) | 7.29 | 5.14 | 0 | 0 | 2.96 | 5.58 | 1.51 | 1.51 |
|  |  | GC content | 0.28 | 0.38 | 0 | 0 | 0.41 | 0.24 | 0 | 0 |
|  | Dinucleotide | No.of SSRs | 9 | 1 | 0 | 0 | 5 | 3 | 1 | 1 |
|  |  | Relative abundance (No./Kb) | 0.17 | 0.07 | 0 | 0 | 0.05 | 0.14 | 0.04 | 0.04 |
|  |  | Relative density (bp/Kb) | 2.52 | 0.92 | 0 | 0 | 0.85 | 2.12 | 0.50 | 0.50 |
|  |  | GC content | 0.06 | 0 | 0 | 0 | 0.10 | 0 | 0 | 0 |
|  | Trinucleotide | No.of SSRs | 2 | 0 | 0 | 0 | 0 | 2 | 0 | 0 |
|  |  | Relative abundance (No./Kb) | 0.04 | 0 | 0 | 0 | 0 | 0.09 | 0 | 0 |
|  |  | Relative density (bp/Kb) | 0.45 | 0 | 0 | 0 | 0 | 1.11 | 0 | 0 |
|  |  | GC content | 0 | 0 | 0 | 0 | 0 | 0 | 0 | 0 |
|  | Tetranucleotide | No.of SSRs | 11 | 1 | 0 | 0 | 7 | 3 | 1 | 1 |
|  |  | Relative abundance (No./Kb) | 0.21 | 0.07 | 0 | 0 | 0.08 | 0.14 | 0.04 | 0.04 |
|  |  | Relative density (bp/Kb) | 2.56 | 0.79 | 0 | 0 | 0.91 | 1.85 | 0.50 | 0.50 |
|  |  | GC content | 0.15 | 0 | 0 | 0 | 0.18 | 0.08 | 0.25 | 0.25 |
|  | Pentanucleotide | No.of SSRs | 1 | 0 | 0 | 0 | 1 | 0 | 0 | 0 |
|  |  | Relative abundance (No./Kb) | 0.02 | 0 | 0 | 0 | 0.01 | 0 | 0 | 0 |
|  |  | Relative density (bp/Kb) | 0.28 | 0 | 0 | 0 | 0.16 | 0 | 0 | 0 |
|  |  | GC content | 0.4 | 0 | 0 | 0 | 0.4 | 0 | 0 | 0 |
|  | Five nucleotides | Total No. of SSRs | 56 | 9 | 0 | 0 | 38 | 17 | 5 | 5 |
| *A. costularis* | mononucleotide | No.of SSRs | 80 | 15 | 0 | 0 | 67 | 16 | 6 | 6 |
|  |  | Relative abundance (No./Kb) | 1.66 | 0.94 | 0 | 0 | 0.78 | 0.74 | 0.25 | 0.25 |
|  |  | Relative density (bp/Kb) | 20.49 | 13.27 | 0 | 0 | 9.78 | 8.83 | 3.41 | 3.41 |
|  |  | GC content | 0.19 | 0.36 | 0 | 0 | 0.20 | 0.19 | 0.36 | 0.36 |
|  | Dinucleotide | No.of SSRs | 13 | 1 | 0 | 0 | 7 | 3 | 2 | 2 |
|  |  | Relative abundance (No./Kb) | 0.27 | 0.06 | 0 | 0 | 0.08 | 0.14 | 0.08 | 0.08 |
|  |  | Relative density (bp/Kb) | 3.89 | 1.50 | 0 | 0 | 1.32 | 2.31 | 0.99 | 0.99 |
|  |  | GC content | 0 | 0 | 0 | 0 | 0 | 0 | 0 | 0 |
|  | Trinucleotide | No.of SSRs | 0 | 1 | 0 | 0 | 1 | 0 | 0 | 0 |
|  |  | Relative abundance (No./Kb) | 0 | 0.06 | 0 | 0 | 0.01 | 0 | 0 | 0 |
|  |  | Relative density (bp/Kb) | 0 | 0.75 | 0 | 0 | 0.14 | 0 | 0 | 0 |
|  |  | GC content | 0 | 0.33 | 0 | 0 | 0.33 | 0 | 0 | 0 |
|  | Tetranucleotide | No.of SSRs | 7 | 1 | 3 | 0 | 6 | 1 | 2 | 2 |
|  |  | Relative abundance (No./Kb) | 0.14 | 0.06 | 0.04 | 0 | 0.07 | 0.05 | 0.08 | 0.08 |
|  |  | Relative density (bp/Kb) | 1.82 | 0.75 | 0.45 | 0 | 0.88 | 0.55 | 0.99 | 0.99 |
|  |  | GC content | 0.22 | 0.75 | 0.33 | 0 | 0.29 | 0.50 | 0.25 | 0.25 |
|  | Pentanucleotide | No.of SSRs | 0 | 0 | 0 | 0 | 0 | 0 | 0 | 0 |
|  |  | Relative abundance (No./Kb) | 0 | 0 | 0 | 0 | 0 | 0 | 0 | 0 |
|  |  | Relative density (bp/Kb) | 0 | 0 | 0 | 0 | 0 | 0 | 0 | 0 |
|  |  | GC content | 0 | 0 | 0 | 0 | 0 | 0 | 0 | 0 |
|  | Five nucleotides | Total No. of SSRs | 99 | 19 | 3 | 0 | 81 | 20 | 10 | 10 |
| *A. spinulosa* | mononucleotide | No.of SSRs | 81 | 15 | 0 | 0 | 68 | 16 | 6 | 6 |
|  |  | Relative abundance (No./Kb) | 1.68 | 0.99 | 0 | 0 | 0.79 | 0.74 | 0.25 | 0.25 |
|  |  | Relative density (bp/Kb) | 20.73 | 12.45 | 0 | 0 | 9.63 | 8.74 | 3.49 | 3.49 |
|  |  | GC content | 0.18 | 0.28 | 0 | 0 | 0.17 | 0.19 | 0.31 | 0.31 |
|  | Dinucleotide | No.of SSRs | 13 | 1 | 0 | 0 | 7 | 3 | 2 | 2 |
|  |  | Relative abundance (No./Kb) | 0.27 | 0.07 | 0 | 0 | 0.08 | 0.14 | 0.08 | 0.08 |
|  |  | Relative density (bp/Kb) | 3.89 | 1.58 | 0 | 0 | 1.32 | 2.31 | 0.99 | 0.99 |
|  |  | GC content | 0 | 0 | 0 | 0 | 0 | 0 | 0 | 0 |
|  | Trinucleotide | No.of SSRs | 0 | 1 | 0 | 0 | 1 | 0 | 0 | 0 |
|  |  | Relative abundance (No./Kb) | 0 | 0.07 | 0 | 0 | 0.01 | 0 | 0 | 0 |
|  |  | Relative density (bp/Kb) | 0 | 0.79 | 0 | 0 | 0.14 | 0 | 0 | 0 |
|  |  | GC content | 0 | 0.33 | 0 | 0 | 0.33 | 0 | 0 | 0 |
|  | Tetranucleotide | No.of SSRs | 7 | 1 | 3 | 0 | 6 | 1 | 2 | 2 |
|  |  | Relative abundance (No./Kb) | 0.14 | 0.07 | 0.04 | 0 | 0.07 | 0.05 | 0.08 | 0.08 |
|  |  | Relative density (bp/Kb) | 1.82 | 0.79 | 0.45 | 0 | 0.88 | 0.55 | 0.99 | 0.99 |
|  |  | GC content | 0.22 | 0.75 | 0.33 | 0 | 0.29 | 0.50 | 0.25 | 0.25 |
|  | Pentanucleotide | No.of SSRs | 0 | 0 | 0 | 0 | 0 | 0 | 0 | 0 |
|  |  | Relative abundance (No./Kb) | 0 | 0 | 0 | 0 | 0 | 0 | 0 | 0 |
|  |  | Relative density (bp/Kb) | 0 | 0 | 0 | 0 | 0 | 0 | 0 | 0 |
|  |  | GC content | 0 | 0 | 0 | 0 | 0 | 0 | 0 | 0 |
|  | Five nucleotides | Total No. of SSRs | 100 | 19 | 3 | 0 | 82 | 20 | 10 | 10 |
| *S. brunoniana* | mononucleotide | No.of SSRs | 62 | 15 | 0 | 0 | 61 | 10 | 3 | 3 |
|  |  | Relative abundance (No./Kb) | 1.29 | 1.04 | 0 | 0 | 0.71 | 0.45 | 0.12 | 0.12 |
|  |  | Relative density (bp/Kb) | 17.20 | 12.25 | 0 | 0 | 9.22 | 5.66 | 1.71 | 1.71 |
|  |  | GC content | 0.08 | 0.08 | 0 | 0 | 0.10 | 0 | 0 | 0 |
|  | Dinucleotide | No.of SSRs | 9 | 6 | 0 | 0 | 10 | 3 | 1 | 1 |
|  |  | Relative abundance (No./Kb) | 0.19 | 0.42 | 0 | 0 | 0.12 | 0.13 | 0.04 | 0.04 |
|  |  | Relative density (bp/Kb) | 2.95 | 5.40 | 0 | 0 | 1.72 | 2.14 | 0.50 | 0.50 |
|  |  | GC content | 0.04 | 0.24 | 0 | 0 | 0.05 | 0.13 | 0.50 | 0.50 |
|  | Trinucleotide | No.of SSRs | 0 | 0 | 0 | 0 | 0 | 0 | 0 | 0 |
|  |  | Relative abundance (No./Kb) | 0 | 0 | 0 | 0 | 0 | 0 | 0 | 0 |
|  |  | Relative density (bp/Kb) | 0 | 0 | 0 | 0 | 0 | 0 | 0 | 0 |
|  |  | GC content | 0 | 0 | 0 | 0 | 0 | 0 | 0 | 0 |
|  | Tetranucleotide | No.of SSRs | 6 | 0 | 2 | 2 | 4 | 0 | 3 | 3 |
|  |  | Relative abundance (No./Kb) | 0.12 | 0 | 0.02 | 0.22 | 0.05 | 0 | 0.12 | 0.12 |
|  |  | Relative density (bp/Kb) | 1.66 | 0 | 0.30 | 2.64 | 0.65 | 0 | 1.50 | 1.50 |
|  |  | GC content | 0.25 | 0 | 0.25 | 0.5 | 0.25 | 0 | 0.33 | 0.33 |
|  | Pentanucleotide | No.of SSRs | 0 | 0 | 0 | 0 | 0 | 0 | 0 | 0 |
|  |  | Relative abundance (No./Kb) | 0 | 0 | 0 | 0 | 0 | 0 | 0 | 0 |
|  |  | Relative density (bp/Kb) | 0 | 0 | 0 | 0 | 0 | 0 | 0 | 0 |
|  |  | GC content | 0 | 0 | 0 | 0 | 0 | 0 | 0 | 0 |
|  | Five nucleotides | Total No. of SSRs | 77 | 21 | 2 | 2 | 75 | 13 | 7 | 7 |
| *S. lepifera* | mononucleotide | No.of SSRs | 69 | 11 | 0 | 0 | 62 | 12 | 3 | 3 |
|  |  | Relative abundance (No./Kb) | 1.28 | 0.75 | 0 | 0 | 0.72 | 0.43 | 0.12 | 0.12 |
|  |  | Relative density (bp/Kb) | 16.43 | 8.63 | 0 | 0 | 9.11 | 5.19 | 1.66 | 1.66 |
|  |  | GC content | 0.07 | 0.24 | 0 | 0 | 0.12 | 0 | 0 | 0 |
|  | Dinucleotide | No.of SSRs | 8 | 3 | 0 | 0 | 5 | 4 | 1 | 1 |
|  |  | Relative abundance (No./Kb) | 0.15 | 0.20 | 0 | 0 | 0.06 | 0.14 | 0.04 | 0.04 |
|  |  | Relative density (bp/Kb) | 2.38 | 2.72 | 0 | 0 | 0.95 | 2.09 | 0.58 | 0.58 |
|  |  | GC content | 0.05 | 0.35 | 0 | 0 | 0 | 0.10 | 0.50 | 0.50 |
|  | Trinucleotide | No.of SSRs | 0 | 0 | 0 | 0 | 0 | 0 | 0 | 0 |
|  |  | Relative abundance (No./Kb) | 0 | 0 | 0 | 0 | 0 | 0 | 0 | 0 |
|  |  | Relative density (bp/Kb) | 0 | 0 | 0 | 0 | 0 | 0 | 0 | 0 |
|  |  | GC content | 0 | 0 | 0 | 0 | 0 | 0 | 0 | 0 |
|  | Tetranucleotide | No.of SSRs | 6 | 0 | 4 | 2 | 3 | 1 | 4 | 4 |
|  |  | Relative abundance (No./Kb) | 0.11 | 0 | 0.05 | 0.22 | 0.03 | 0.04 | 0.17 | 0.17 |
|  |  | Relative density (bp/Kb) | 1.41 | 0 | 0.60 | 2.64 | 0.46 | 0.43 | 1.99 | 1.99 |
|  |  | GC content | 0.17 | 0 | 0.13 | 0.50 | 0.18 | 0 | 0.25 | 0.25 |
|  | Pentanucleotide | No.of SSRs | 0 | 0 | 0 | 0 | 0 | 0 | 0 | 0 |
|  |  | Relative abundance (No./Kb) | 0 | 0 | 0 | 0 | 0 | 0 | 0 | 0 |
|  |  | Relative density (bp/Kb) | 0 | 0 | 0 | 0 | 0 | 0 | 0 | 0 |
|  |  | GC content | 0 | 0 | 0 | 0 | 0 | 0 | 0 | 0 |
|  | Five nucleotides | Total No. of SSRs | 83 | 14 | 4 | 2 | 70 | 17 | 8 | 8 |

Appendix Table S11 Significant differences in the number, relative abundance, relative density, and GC content of the chloroplast genomes, mono- to pentanucleotide SSRs, IGS, LSC, intronic and CDS regionsSSRs of chloroplast genomes, and the mono- to pentanucleotide SSRs in the chloroplast genomes of eight Cyatheaceae species

| Classification treatment | Characteristics of SSR | P | | | | | | | | | | | | | |
| --- | --- | --- | --- | --- | --- | --- | --- | --- | --- | --- | --- | --- | --- | --- | --- |
|  |  | Genome | Mono- | Di- | Tri- | Tetra- | Penta- | IGS | intron | CDS | rRNA gene | LSC | SSC | IRA | IRB |
| 3 genera | No.of SSRs | 0.048* | 0.048* | 0.065 | 0.057 | 0.836 | 0.122 | 0.048* | 0.067 | 0.046* | 0.030* | 0.048* | 0.118 | 0.046* | 0.046* |
|  | Relative abundance (No./Kb) | 0.033* | 0.050* | 0.069 | 0.103 | 0.400 | 0.140 | 0.050* | 0.069 | 0.048* | 0.030* | 0.050* | 0.127 | 0.050* | 0.050* |
|  | Relative density | 0.050* | 0.050* | 0.069 | 0.103 | 0.673 | 0.140 | 0.050* | 0.069 | 0.048* | 0.030* | 0.050* | 0.135 | 0.050* | 0.050* |
|  | GC content | 0.050* | 0.050* | 0.068 | 0.030* | 0.065 | 0.311 | 0.050* | 0.264 | 0.032* | 0.030* | 0.050* | 0.130 | 0.050* | 0.050* |
| 2 genera | No.of SSRs | 0.502 | 0.502 | 0.177 | 0.034* | 0.729 | 0.237 | 0.502 | 0.180 | 0.153 | 0.008* | 0.502 | 0.734 | 0.500 | 0.500 |
|  | Relative abundance (No./Kb) | 0.478 | 0.505 | 0.182 | 0.044* | 0.505 | 0.252 | 0.505 | 0.182 | 0.155 | 0.008* | 0.505 | 0.317 | 0.505 | 0.505 |
|  | Relative density | 0.505 | 0.505 | 0.182 | 0.044* | 0.739 | 0.252 | 0.505 | 0.182 | 0.155 | 0.008* | 0.505 | 0.505 | 0.505 | 0.505 |
|  | GC content | 0.046* | 0.046* | 0.040* | 0.378 | 0.177 | 0.378 | 0.046* | 0.182 | 0.475 | 0.008* | 0.046* | 0.044* | 0.505 | 0.505 |
|  |  |  |  |  |  |  |  |  |  |  |  |  |  |  |  |
| P | | | | | | | | | | | | | | | |
| IGS-Mono | intron-Mono | CDS-Mono | rRNA gene-Mono | LSC-Mono | SSC-Mono | IRA-Mono | IRB-Mono | IGS-Di | intron-Di | CDS-Di | rRNA gene-Di | LSC-Di | SSC-Di | IRA-Di | IRB-Di |
| 0.048 | 0.056 | 1 | 1 | 0.048 | 0.046* | 0.112 | 0.112 | 0.114 | 0.032* | 1 | 1 | 0.108 | 0.269 | 0.030* | 0.030* |
| 0.050* | 0.069 | 1 | 1 | 0.050* | 0.050* | 0.135 | 0.135 | 0.105 | 0.127 | 1 | 1 | 0.069 | 0.293 | 0.105 | 0.105 |
| 0.050* | 0.050* | 1 | 1 | 0.050* | 0.050* | 0.050* | 0.050* | 0.105 | 0.050* | 1 | 1 | 0.069 | 0.408 | 0.127 | 0.127 |
| 0.050* | 0.223 | 1 | 1 | 0.050* | 0.103 | 0.067 | 0.067 | 0.353 | 0.248 | 1 | 1 | 0.206 | 0.032* | 0.030* | 0.030* |
| 0.502 | 0.306 | 1 | 1 | 0.502 | 0.500 | 0.731 | 0.731 | 0.604 | 0.009* | 1 | 1 | 0.299 | 0.127 | 0.378 | 0.378 |
| 0.505 | 0.182 | 1 | 1 | 0.505 | 0.505 | 0.505 | 0.505 | 1 | 0.046* | 1 | 1 | 0.182 | 0.739 | 0.182 | 0.182 |
| 0.505 | 0.505 | 1 | 1 | 0.505 | 0.739 | 0.505 | 0.505 | 1 | 0.046* | 1 | 1 | 0.182 | 0.739 | 0.739 | 0.739 |
| 0.046 | 0.096 | 1 | 1 | 0.046* | 0.044* | 0.252 | 0.252 | 0.478 | 0.127 | 1 | 1 | 0.594 | 0.009* | 0.008* | 0.008* |
|  |  |  |  |  |  |  |  |  |  |  |  |  |  |  |  |
| P | | | | | | | | | | | | | | | |
| IGS-Tri | intron-Tri | CDS-Tri | rRNA gene-Tri | LSC-Tri | SSC-Tri | IRA-Tri | IRB-Tri | IGS-Tetra | intron-Tetra | CDS-Tetra | rRNA gene-Tetra | LSC-Tetra | SSC-Tetra | IRA-Tetra | IRB-Tetra |
| 0.122 | 0.122 | 1 | 1 | 0.122 | 0.122 | 1 | 1 | 0.048* | 0.030* | 0.046* | 0.030* | 0.032* | 0.063 | 0.041* | 0.041* |
| 0.14 | 0.140 | 1 | 1 | 0.067 | 0.14 | 1 | 1 | 0.105 | 0.124 | 0.048* | 0.030* | 0.050* | 0.050* | 0.050* | 0.050* |
| 0.14 | 0.140 | 1 | 1 | 0.067 | 0.14 | 1 | 1 | 0.223 | 0.124 | 0.048* | 0.030* | 0.050* | 0.050* | 0.050* | 0.050* |
| 1 | 0.030* | 1 | 1 | 0.030* | 1 | 1 | 1 | 0.160 | 0.030* | 0.032* | 0.030* | 0.129 | 0.075 | 0.269 | 0.269 |
| 0.237 | 0.237 | 1 | 1 | 0.237 | 0.237 | 1 | 1 | 0.044* | 0.008* | 0.153 | 0.008* | 0.032* | 0.038* | 0.039* | 0.039* |
| 0.252 | 0.252 | 1 | 1 | 0.252 | 0.252 | 1 | 1 | 0.046* | 0.044* | 0.155 | 0.008* | 0.046* | 0.046* | 0.046* | 0.046* |
| 0.252 | 0.252 | 1 | 1 | 0.252 | 0.252 | 1 | 1 | 0.317 | 0.044* | 0.155 | 0.008* | 0.046* | 0.044* | 0.046* | 0.046* |
| 1 | 0.378 | 1 | 1 | 0.378 | 1 | 1 | 1 | 0.739 | 0.378 | 0.475 | 0.008* | 0.500 | 0.153 | 0.127 | 0.127 |
|  |  |  |  |  |  |  |  |  |  |  |  |  |  |  |  |
| P | | | | | | | |  |  |  |  |  |  |  |  |
| IGS-Penta | intron-Penta | CDS-Penta | rRNA gene-Penta | LSC-Penta | SSC-Penta | IRA-Penta | IRB-Penta |  |  |  |  |  |  |  |  |
| 0.311 | 0.607 | 1 | 1 | 0.122 | 1 | 1 | 1 |  |  |  |  |  |  |  |  |
| 0.319 | 0.607 | 1 | 1 | 0.140 | 1 | 1 | 1 |  |  |  |  |  |  |  |  |
| 0.319 | 0.607 | 1 | 1 | 0.140 | 1 | 1 | 1 |  |  |  |  |  |  |  |  |
| 0.607 | 0.607 | 1 | 1 | 0.311 | 1 | 1 | 1 |  |  |  |  |  |  |  |  |
| 0.378 | 0.564 | 1 | 1 | 0.237 | 1 | 1 | 1 |  |  |  |  |  |  |  |  |
| 0.383 | 0.564 | 1 | 1 | 0.252 | 1 | 1 | 1 |  |  |  |  |  |  |  |  |
| 0.383 | 0.564 | 1 | 1 | 0.252 | 1 | 1 | 1 |  |  |  |  |  |  |  |  |
| 0.564 | 0.564 | 1 | 1 | 0.378 | 1 | 1 | 1 |  |  |  |  |  |  |  |  |

When 8 Cyatheaceae plant species are categorized into as three genera, the Kruskal-Wallis H test is used; when they are categorized into two genera, the Mann-Whitney U test is used; *：*P*≤0.05; IGS: Intergenic spacer-region; LSC: Large single copy-region; CDS:coding sequence-region
